# Microalgae biotechnology and its role in sustainable and healthy food design

**DOI:** 10.3389/fbioe.2025.1716473

**Published:** 2025-12-10

**Authors:** Adriane Terezinha Schneider, Richard Luan Silva Machado, Darissa Alves Dutra, Eduarda Funari Machado, Rosangela Rodrigues Dias, Mariany Costa Deprá, Leila Queiroz Zepka, Eduardo Jacob-Lopes

**Affiliations:** Bioprocess Intensification Group, Federal University of Santa Maria, UFSM, Santa Maria, Brazil

**Keywords:** microalgae, food regulation, functional foods, consumer acceptance, sustainability

## Abstract

Current food systems face a paradox: although scientific and technological advances have increased production capacity, they still cannot ensure nutritious and sustainable diets for everyone worldwide. In this context, microalgae stand out as promising bioresources due to their nutritional value, functional properties, and environmental benefits. This review critically examines the current state of microalgae biotechnology for food applications, focusing on cultivation methods, processes, techno-functional properties, regulatory challenges, and consumer perceptions. The analysis indicates that, despite notable progress in cultivation systems and approaches to integration and intensification, high production costs and inconsistent methods of characterizing microalgal biomass remain major obstacles to limit large-scale competitiveness. Additionally, legislation and consumer acceptance issues create a gap between laboratory innovations and industrial implementation. To make microalgae a mainstream ingredient, it is essential: (i) align safety standards and regulations; (ii) incorporate economic feasibility and sustainability; and (iii) develop strategic approaches that translate scientific advancements into practical consumer benefits. Therefore, this study, which explores the intersection of biotechnology, nutrition, and economics, offers a valuable framework to help turn microalgae from a promising idea into a practical solution within global food systems.

## 1 Introduction

Food plays such an essential role in human existence that we rarely stop to reflect on its true purpose. It constitutes the core substrate for life, transcending the mere provision of energy and nutrients to assume complex and multifaceted functions in the social, cultural, and economic spheres (Gowder, 2024). In addition to nourishing, foods act as vectors of interaction, celebration, and strengthening of social bonds, as a structuring element of individual and collective health and wellbeing, intrinsically reflecting socioeconomic dynamics and the management of natural resources in contemporary society (Andersen and Hyldig, 2015).

This centrality of foods in human life has driven - throughout history–profound transformations in the form of food products. From the agricultural consolidation—more than 10,000 years ago—along with the technological advances of the Industrial Revolution, humanity has expanded its sustenance capacity and built social systems capable of increasingly intervening in global ecosystems (Funabashi, 2018). However, these achievements are followed by significant environmental paradoxes, such as the intensified of agricultural production, intensive use of synthetic pesticides, as well as the high demand for energy and the exploration of non-renewable resources (Khatri et al., 2024).

These historical challenges shaped the contemporary scenario; food systems, instead of fully solving the problems of access and sustainability, face even more complex pressures. Currently, climate change, population growth, and resource scarcity intensify the risks to human health, collective welfare, environmental quality, and socioeconomic development. Global estimates indicate that, in 2020, approximately 2.37 billion people lacked access to an adequate diet, and nearly 11% of the world’s population suffered from malnutrition—characterized by deficiencies in vitamins, minerals, and macronutrients (Moura et al., 2023). In 2022, around 695 million people faced hunger (FAO, 2025). Last year, this figure remained alarming, with 673 million individuals—representing about 8.2% of the global population—according to the World Health Organization (2025). Although the number of undernourished people worldwide is expected to decline, projections still estimate that by 2030, approximately 512 million people will face hunger (FAO, 2025). This scenario underscores not only the urgency of adopting new approaches but also the inefficiency of current food systems in ensuring global nutritional security.

In response to these challenges, interest in and development of innovative and sustainable food solutions are increasing, particularly in underexplored sources, including plants, fungi, and microalgae, which hold significant potential to address the nutritional requirements of the world’s population (Mariutti et al., 2021). Thus, microalgae emerge as promising alternatives, gaining prominence due to the intersection between biotechnology-sustainability-nutrition. Their biomass contains high levels of protein (30%–70% of dry matter) and is also an important source of essential amino acids, polyunsaturated fatty acids, and high-value-added metabolites, such as β-carotene, astaxanthin, and lutein, which are widely recognized for their bioactive properties and health promotion (Oliveira et al., 2022). In addition to their unique composition, microalgae cultivation generally does not compete with arable land and can contribute to carbon capture, positioning microalgae as a strategic tool for both climate change mitigation and the circular bioeconomy.

Among the most commercially relevant species, *Chlorella sp*. and *Arthrospira sp* (commercially known as *Spirulina*) are widely distributed in the productive sector by virtue of their high growth rate, notable adaptability to different environmental conditions, and valuable nutritional composition. The use of *Arthrospira* sp. dates back to ancient practices of indigenous people in different regions of the world—such as Peru, China, Mexico, and Japan—where it was consumed as an essential food source. In today’s context, these species are valued not only as a nutritional supplement but also for its potential in preventing diseases and promoting health (Lu et al., 2011; Pérez-Lloréns, 2020; Çelekli et al., 2024). *Chlorella* sp., in turn, is recognized by the Food and Agriculture Organization (FAO) as a “*healthy green food*”, and has been consolidated as one of the most widely produced microalgae on a large scale in Europe, Asia, and the United States. Its application is especially intended for the sector of dietary supplements, nutraceuticals, and pharmaceutical formulations (Saurav et al., 2019; Yuan et al., 2020; Sinetova et al., 2024).

The advances in knowledge on the nutritional and bioactive value of species such as *Chlorella* sp. and *Arthrospira* sp. have had repercussions in the economic sphere, translating into greater commercial acceptance and diversification of applications. Thus, current market trends consolidate this growing interest. Throughout the 5-year period from 2014 to 2019, the market share of products containing microalgae increased exponentially, reflecting its role as an innovative and sustainable ingredient in food fortification (Lafarga, 2019; Refaey et al., 2024). However, hurdles still exist in terms of their wide use on a large scale and their economic viability when compared to conventional food ingredients. For instance, the market price of basic inputs widely used in the industry, such as cassava starch, corn starch, soybean meal, refined sugar, and salt, is relatively stable and affordable, whereas microalgal biomass has a significantly higher market price (Caporgno and Mathys, 2018). This discrepancy in market price is represented in Table 1, which shows the contrast between conventional and emerging sources, demonstrating the limited competitiveness of microalgae in the food sector.

**TABLE 1:** Overview of the market price of ingredients in the food industry.

Ingredient	Market price	References
*Conventional ingredients*		
Cassava starch	909.00 USD/t of dry biomass^a^	IMARC Group (2025a)
Corn starch	496.00 USD/t of dry biomass^a^	IMARC Group (2025b)
Sodium chloride	220.00 USD/t of dry biomass^b^	Tridge (2025)
Soybean meal	313.11 USD/t of dry biomass^c^	International Monetary Fund (2025)
White sugar	478.45 USD/t of dry biomass^c^	Isosugar (2025)
*Microalgae biomass*		
*Arthrospira*	299,500.00 USD/t of dry biomass^b^	Royal spirulina (2025)
*Chlorella*	124,495.00 USD/t of dry biomass^b^	Allmicroalgae (2025)

^a^ Referring to the first quarter of 2025.

^b^ Referring to the year 2025.

^c^ Referring to June/2025.

Despite the high nutritional and functional value of microalgal biomass and increasing interest in its application as a sustainable ingredient, its effective insertion as a conventional food component is still subject to structural and economic barriers. The main hurdles are associated with the transition from laboratory cultivation to an industrial scale that frequently results in losses of productivity, difficulties in controlling variables, and limitations in the efficiency of cultivation systems, evidencing the obstacles and complexities of scalability (Deprá et al., 2025). In fact, the differences observed between laboratory and industrial-scale processes are largely attributed to the sensitivity of microalgae performance to critical operational parameters. Factors such as light intensity and photoperiod, CO_2_ concentration and distribution efficiency, nutrient availability, and mixing or agitation systems behave differently in controlled laboratory environments compared to large-scale cultures. Consequently, variations in these parameters lead to substantial differences in growth rates, biomass yield, and metabolite accumulation, thereby widening the performance gap between laboratory and industrial operations (Caporgno and Mathys, 2018; Deprá et al., 2024; Guieysse and Plouviez, 2024). Associated with this, production costs remain high, mainly due to the harvesting and drying stages, which may represent most of the final product value (Caporgno and Mathys, 2018). Technologically, the risks of contamination, biofilm formation in photobioreactors, inadequate light distribution, shear stress, and limited temperature control compromise not only productivity but also industrial viability (Deprá et al., 2024).

Additionally, technological and economic challenges do not occur in isolation; these combine with equally critical external factors, such as the need for regulatory approval and market acceptance, which directly influence the viability and expansion of the microalgae use in conventional foods. In this context, a combination of high costs, regulatory complexity, and consumer perception positions microalgae at an ambiguous borundary between food and medicinal applications—especially in encapsulated or nutraceutical forms—making it difficult to include them on a large-scale in conventional diets (Guil-Guerrero and Prates, 2025; Hosseinkhani et al., 2022). Between promises and bottlenecks, the use of microalgae in food is configured as a space of opportunities that can only be fully explored as science, technology, and the market advance in synergy (Caporgno and Mathys, 2018). This scenario, therefore, shows a paradox: although recognized for their nutritional value and sustainability, microalgae have not yet consolidated on a large-scale in food systems.

Given this background, this article proposes a critical and integrated analysis of the potential microalgae as catalysts of innovation in the design of functional and sustainable foods. The focus of this review is to consolidate the most recent scientific advances, while systematically examining the challenges that limit large-scale adoption of these microorganisms, including technological limitations and economic barriers, regulatory restrictions, and consumer perception and acceptance; factors that directly influence the viability of their integration into conventional food systems. Here, we seek to highlight strategic opportunities that allow us to fully explore the potential of microalgae as the next-generation of food design. Understanding the trajectory of microalgae in the food sector, therefore, becomes essential to anticipate market trends, guide regulatory policies, and promote sustainable industrial practices that transform emerging promises into concrete solutions. By emphasizing the interconnection between science, technology, and the market, this article seeks to provide critical insights for researchers, product developers, and public policy managers, strengthening understanding of how to maximize the potential of these species and consolidate them as pivotal elements of an innovative, sustainable food bioeconomy capable of contributing to nutritional and environmental safety on a global scale.

## 2 Microalgae cultivation systems: charting opportunities and overcoming bottlenecks for innovative food design

The starting point for the success of microalgae as a promising food source is directly linked to cultivation systems (de Oliveira and Bragotto, 2022). Although the selection of the strain and its biological potential is crucial for defining the nutritional and functional quality of the bioproducts, it is in the cultivation strategies that the decisive factor for large-scale process viability is found. After all, without an efficient structure to multiply biomass in commercial volumes, even the most potent strains remain confined to the laboratory environment (Mutanda et al., 2020).

As efforts to scale up microalgae production for food applications progress, it becomes essential to understand the different technological approaches available for cultivation in an industrial setting (Liu et al., 2023). In general, these systems can be grouped into three main categories: open systems, closed systems (photobioreactors), and hybrid systems. Each one has specific characteristics in terms of physical structure, operational control, and economic feasibility, directly influencing process performance, production efficiency, and environmental sustainability (Razzak et al., 2024). PBRs offer advantages in terms of productivity. In open systems, typical biomass productivity concentrations are approximately 0.12–0.48 g L^-1^day^-1^. In contrast, PBRs typically achieve higher productivity concentrations, ranging from 0.2 to 3.8 g L^-1^day^-1^ (Francke et al., 2022; Shekh et al., 2022; Skifa et al., 2024).

**FIGURE 1:**
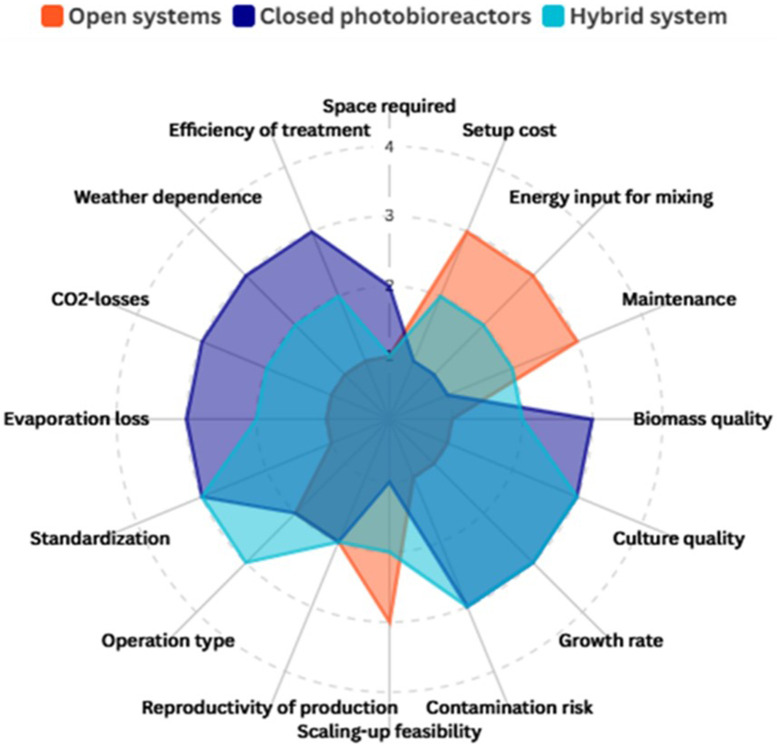
Comparative parameters of cultivation systems: 1 = unfavorable performance/limiting conditions; 2 = intermediate/moderate performance; 3 = favorable performance/advantageous conditions.

Choosing the most suitable cultivation system, however, should not be based on a single criterion. The efficient production of microalgae-based bioproducts involves a series of interdependent variables, such as operational cost, yield per unit area, control of environmental variables, energy consumption, and environmental impact throughout the life cycle of the process (Novoveská et al., 2023). Considering these dimensions in an integrated way is essential to ensure not only productivity but also sustainability and economic competitiveness of the final product (Biswas et al., 2025). Therefore, to guide this comparative assessment, Figure 1 presents a summary of the main advantages and limitations associated with each type of cultivation system, serving as a basis for strategic decisions regarding the development and scaling of biotechnological processes with microalgae.

**FIGURE 2:**
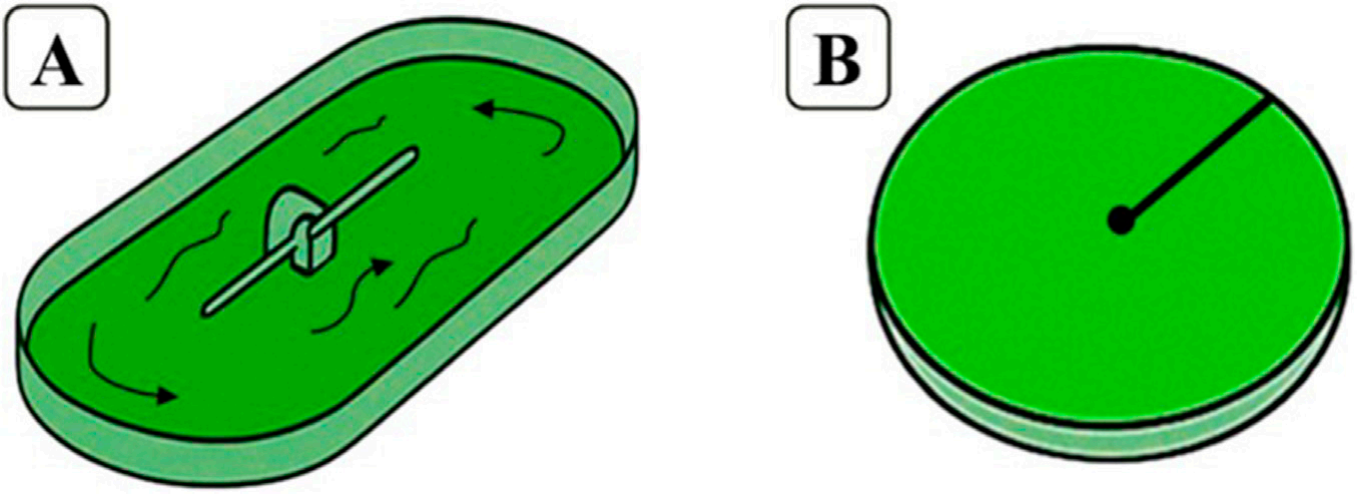
Illustrations of examples of open systems: **(A)** raceway ponds; **(B)** circular ponds.

Among these, open systems cultivation stands out. Although historically the most widely used due to their structural simplicity and low initial costs, they face significant limitations when considered for large-scale applications, particularly in the food sector. Open cultivation systems is among the most conventional and economically accessible methods for microalgae production, often implemented in regions with available land and favorable climatic conditions (De Andrade et al., 2022; Zhang et al., 2024). Their operation is mainly based on structures such as raceway ponds, circular ponds, and similar configurations (Figure 2), which exhibit a high surface-to-volume (S/V) ratio due to the shallow depth of the medium. This characteristic enhances light penetration and gas exchange but also results in low volumetric productivity, thereby requiring large land areas—often between 5 and 10 m^2^ per production unit (de Oliveira Prado et al., 2023). Despite their simplicity, these systems present critical operational disadvantages, including high evaporation rates, significant CO_2_ losses through degassing, and high energy consumption to maintain continuous mechanical mixing, which is essential to prevent sedimentation and ensure culture uniformity. Furthermore, they are highly susceptible to biological contamination, climatic fluctuations, and environmental imbalances, making the process less stable and more vulnerable to external variability. Consequently, the implementation of these systems must account for both extrinsic and intrinsic control factors, since the shallow depth and large surface area required by open ponds create trade-offs between productivity, contamination risk, and land use (Box 1). Additionally, continuous exposure to direct sunlight and the atmosphere further contributes to fluctuations in pH and salinity, negatively affecting the metabolic performance of microalgae (De Andrade et al., 2022). Thus, although open systems offer advantages in terms of initial scalability and reduced investment, their use requires caution, particularly when high productivity, biomass purity, or food-grade applications under strict regulatory standards are desired.

Box 1 **Area-volume relationship in open systems: height and dispersion limits.**

Commercial ponds typically range in depth from 20 to 30 cm (Borowitzka and Vonshak, 2017). This shallow depth offers a high S/V ratio (high light area per unit volume), facilitating solar penetration. However, it requires extensive soil use (spreading) to achieve large volumes. One of the challenges of these systems is that the illuminated volume is limited to just the top few centimeters of the water body, leaving the remaining water volume underutilized. Furthermore, the shallow surface reduces the vertical gradient for gas exchange, making it difficult to add CO_2_, resulting in low dissolved gas concentrations.

Open systems with a high surface/volume ratio (S/V), typically shallow, partially bypass this dark valley. The large surface area relative to the volume allows light to reach nearly all cells near the surface, increasing productivity per unit area. However, increasing volume while maintaining a high (S/V) ratio requires a large exposed area. This makes these systems susceptible to evaporation and contamination, in addition to the fact that depth is constrained by inefficient horizontal mixing.

To address these challenges, protective covers can be implemented to minimize evaporation and prevent contaminant ingress. Furthermore, optimizing the mixing process can ensure homogeneity even at moderate depths. These approaches illustrate the tradeoff between maximizing light exposure and maintaining culture integrity.

In parallel, closed systems—particularly photobioreactors (PBRs)—represent a significant technological advancement compared to the operational limitations of open cultivation systems. PBRs enable highly controlled cultivation conditions, a critical factor for ensuring reproducibility, physiological stability of microalgae, and superior biomass quality. These features make closed systems especially suitable for applications requiring a high degree of standardization and safety, such as the production of functional foods, nutritional supplements, and high-value bioactive ingredients (Penloglou et al., 2024; Ferreira et al., 2025).

**FIGURE 3:**
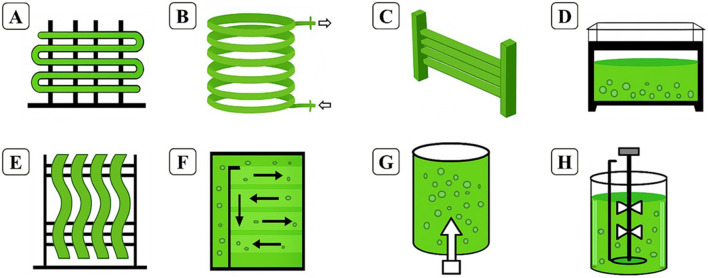
Illustration of different PBRs for microalgae cultivation: **(A)** fence tubular; **(B)** helical tubular; **(C)** horizontal tubular; **(D)** vertical flat panel; **(E)** accordion type; **(F)** air lift type; **(G)** bubble column; **(H)** stirred tank.

One of the main advantages of PBRs is the diversity of available configurations, which allows the system to be adapted to the specific needs of the biotechnological process. Among the most commonly used designs are fence-like tubular photobioreactors, helical tubular reactors, horizontal tubular reactors, vertical flat panels, accordion tubular reactors, air-lift systems, bubble columns, and stirred tanks, illustrated in Figure 3. Each configuration is engineered to address specific bottlenecks related to photosynthesis, such as optimizing incident light, enhancing gas transfer efficiency, maintaining medium homogeneity, and reducing dead zones within the culture (Ranganathan et al., 2022; Shaikh et al., 2023).

It is important to highlight that the different geometries and configurations of the PBRs exert a direct influence on the operational efficiency of microalgae cultures, presenting specific advantages and limitations that must be considered carefully during the planning of processes on an industrial scale. Tubular photobioreactors, for example, are widely used due to their high surface/volume (S/V) ratio, which favors light capture and, consequently, photosynthesis in environments with restricted physical space; However, they are subject to operational challenges, such as the accumulation of dissolved oxygen, the formation of biofilms (biofouling), and the difficulty of internal cleaning, as well as presenting high maintenance costs (Corrêa et al., 2025). In many cases, the materials used in the fabrication of these systems must exhibit a controlled degree of surface roughness to promote cell deposition, thereby ensuring an adequate retention time for the photosynthetic process (Zhang et al., 2020). However, this characteristic can also intensify biofouling, an undesirable aspect of cultivation, as it compromises light penetration, accelerates material degradation, affects culture quality, and increases susceptibility to contamination. Therefore, material selection should balance optical, mechanical, and biological properties. Glass, for instance, exhibits high transparency and excellent light transmittance, in addition to low surface roughness, which initially makes it an attractive material due to reduced biofilm formation and enhanced photosynthetic efficiency. Nevertheless, its low cell adhesiveness, high cost, and structural fragility limit its large-scale application. Polycarbonate, on the other hand, represents a promising alternative, as it combines good light transmittance, high mechanical strength, and lower susceptibility to biofouling, while also offering a more favorable cost–benefit ratio for photobioreactor construction (Deprá et al., 2024). Flat-plate photobioreactors feature homogeneous light distribution along the culture surface, favoring uniformity in cell growth and production of metabolites of interest; Meanwhile, they require a larger installation area and have high structural cost, being more suitable for high added value applications (Rahman and Hellgardt, 2025). The bubble columns are simple vertical systems, with constructed with materials compatible with biological processes and operated by injection mixtures of air and carbon dioxide by means of distributors located at the base, allowing a good homogenization with low mechanical impact on the cells (O’Higgins et al., 2025). Likewise, air-lift systems use pneumatic circulation to promote efficient mixing and reduce hydrodynamic volume, offering an advantageous balance between energy efficiency and cellular integrity, especially in cultures of more sensitive strains (Guler et al., 2025). The agitated tanks and accordion-type reactors, in turn, offer greater mechanical robustness and control over the homogeneity of the mixture, requiring more energy for agitation and having higher operating costs. On the other hand, PBRs made with plastic bags emerge as an alternative with low initial cost and rapid implementation, being advantageous in experimental or small-scale contexts, but limited by low durability of materials, vulnerability to physical damage, and difficulty of scalability (Kishi and Takayama, 2025).

The selection of the most suitable type of photobioreactor must be guided by a multifactorial analysis, which simultaneously considers technical, economic, and operational criteria. Among the main factors to be evaluated is the type of stress desired to stimulate specific metabolic changes (such as high light intensities, nutritional restrictions, or variations in salinity), the productive objectives of the cultures (for example, accumulation of lipids, carotenoids, proteins or other metabolites of interest), as well as restrictions related to infrastructure, available equipment, and local environmental conditions (Sun et al., 2018). Additionally, the geometric parameters of the bioreactor—such as height, diameter, tube compression, and useful volume—exert a decisive influence on several critical aspects of the process, such as efficient light exposure, gas transfer (CO_2_ and O_2_), and homogeneity of the medium (de Mello et al., 2024). Inadequate sizing of these factors can compromise both the technical performance of the system and the economic viability of operation on an industrial scale (Novoveská et al., 2023) (Box 2).

Box 2 **Relationship between height, diameter, and volume: Is there an ideal system?**

The geometry of biological reactors directly influences the volume, illumination, and homogenization of the culture. In a cylindrical reactor, the volume is given as described in Equation 1.

$$V=\pi \times {\left(\frac{D}{2}\right)}^{2}\times H$$ (1)

where *V* is the reactor volume (m^3^), *D* is the cylinder diameter (m), and *H* is the cylinder height (m).

To maintain constant volume, height and diameter are related by Equation 2:

$$H=\frac{4V}{{\pi D}^{2}}$$ (2)

Reducing D requires increasing H, thus raising the height/diameter (H/D) ratio. For example, a 1 m^3^ reactor with D = 0.5 m requires H ≈ 5.1 m (H/D = 10), while with D = 1.0 m, H drops to 1.3 m (H/D = 1.3). Narrow columns (low D, high H/D) increase light exposure per unit volume but face structural limitations. Greater heights improve mass transfer and gas residence time, and the higher area-to-volume ratio can optimize photosynthesis. However, tall columns complicate homogenization, increase operating costs, hydrostatic pressure, and gas solubility effects. In practice, microalgal PBRs adopt high H/D (≥3–5) for illumination efficiency, while conventional fermenters use lower H/D (1:2–1:4) for easier clarification and reduced pumping effort.

At the same time, maximizing the surface/volume (S/V) ratio is crucial to optimizing light capture in microalgae production systems. Narrow internal diameters (≤60 mm) can achieve an S/V ratio of approximately 67 m^2^/m^3^, while a 1 m diameter column has an S/V ratio of only about 6 m^2^/m^3^. Although narrow tubes promote better light penetration, they also present challenges, such as increased clogging risks, increased pumping demands, and increased maintenance complexity. Helical or serpentine designs can expand the illuminated area but tend to be more expensive, susceptible to fouling, and harder to scale. Tall reactors can create light and temperature gradients, while open systems require shallow water (≤30 cm), requiring significantly larger land areas. In this sense, it is possible to consider some sizing examples for industrial production:

In an open pond equipped with a drainage channel (with a depth of 0.3 m and a typical S/V ratio of approximately 10 m^2^/m^3^), the production of 1,000 m^3^ of microalgae biomass would require approximately 3,333 m^2^ of land (0.3 m × area × 1 volume). This reflects low productivity per unit area. In contrast, cylindrical PBRs with a diameter of 0.5 m and a height of 5.1 m achieve an S/V ratio of approximately 67 m^2^/m^3^. The same 1,000 m^3^ volume can therefore be accommodated in a much smaller area of approximately 400 m^2^, thanks to the high column height and favorable S/V ratio, which allow for greater biomass density and better light utilization.

These considerations highlight several trade-offs: a higher H/D and S/V ratio improves illumination and productivity, they also increase engineering complexity and pumping requirements. On the other hand, open systems require significantly more land due to their shallow depths and lower volumetric productivity. Ultimately, there is no one-size-fits-all geometry; reactor design must strike a balance between illumination, mass transfer, mixing efficiency, engineering constraints, and economic viability.

**FIGURE 4:**
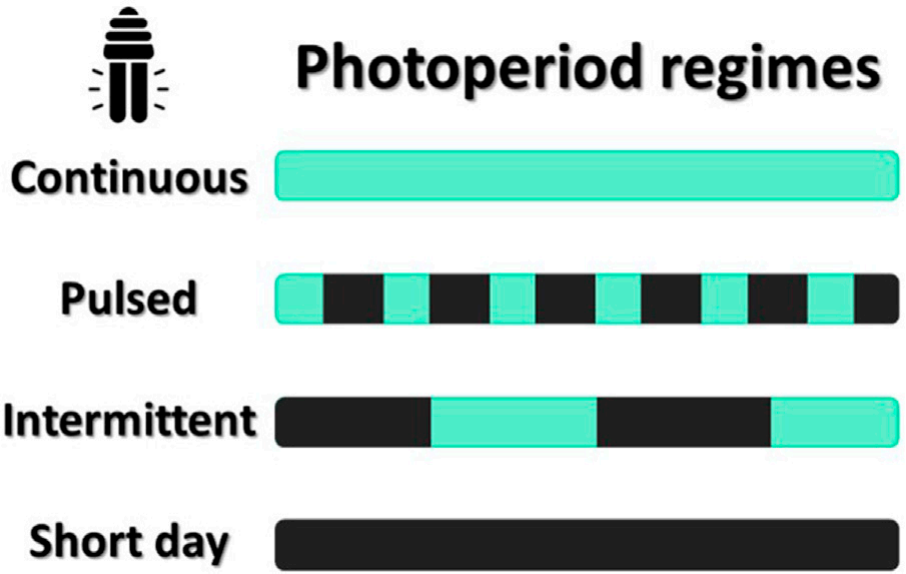
Illustration of photoperiod regimes applicable to microalgae cultivation using LEDs: Continuous–continuous lighting, without periods of darkness; Pulsed–rapid alternation between light and dark at high frequency; Intermittent–long cycles of interspersed light and darkness; and short day–reduced photoperiods, with a predominance of darkness.

Despite the high performance of PBRs under laboratory and pilot-scale conditions, the transition to commercial scales often reveals significant practical limitations. Among the main challenges are heterogeneous light distribution, cellular self-shading, and increased operational costs associated with continuous artificial lighting, particularly in high-cell-density systems. To mitigate these bottlenecks, recent advances in light-emitting diode (LED) technology have shown great promise. This technology not only offers greater energy efficiency but also allows precise control of the spectral and temporal aspects of light. Such control allows the simulation of a variety of photoperiod regimes, including continuous, pulsed, intermittent, and short-day cycles (Borella et al., 2022; Kusuma et al., 2020). In studies with microalgae biofilms, second-scale photoperiods (e.g., 3:3s and 5:5s light/dark) increased biomass yield by ∼11–24% and lipid content by ∼7–22% compared to continuous lighting (Zhang et al., 2019). Minhas et al. (2023), using *Nannochloropsis oculata* grown separately for 23 days under a 13:11 h light/dark photoperiod regime, combined with a light intensity of 250 μE m^-2^ s^-1^, resulted in a lutein productivity of approximately 0.279 mg.L^-1^. day^-1^ and higher lipid productivity when compared to regimes with lower intensity and a 16:8 h photoperiod. Vendruscolo et al. (2019) demonstrated that *Scenedesmus obliquus* grown under a 12:12 light/dark photoperiod had 34% higher lipid content (23.0%) and 20% more total chlorophylls (26.4 mg/g) compared to continuous lighting, although continuous light favored protein accumulation (47.3%). This flexibility is crucial for studying the physiological and biochemical responses of microalgae to different lighting strategies, as illustrated in Figure 4. Furthermore, the ability to implement high-frequency light/dark alternations or intermittent lighting can significantly reduce electricity consumption while maintaining or even improving photosynthetic performance. Therefore, the use of LEDs decouples cultivation from climate variability, enabling continuous and standardized production throughout the year (Kuech et al., 2023).

Another strategic approach to overcome the limitations of phototrophic systems is the use of heterotrophic cultivation in closed bioreactors, in which light is replaced by organic carbon sources as the primary energy input. This mode allows substantially higher cell densities (50–100 g L^-1^) compared to phototrophic systems (2–3 g L^-1^), significantly reducing downstream costs and land requirements (Barros et al., 2019; Glencross et al., 2025). Companies such as Corbion and DSM-Veramaris already employ large-scale fermenters (over 600 m^3^) for the commercial production of omega-3 oils and other high-value compounds (Barros et al., 2019). However, this strategy also requires careful feasibility assessment, particularly regarding feedstock costs, product selectivity, and environmental impacts throughout the life cycle, when compared with phototrophic cultivation routes. To better illustrate these environmental implications, preliminary life cycle assessments of Phormidium autumnale cultivated heterotrophically reported a global warming potential of 171.05 kg CO_2_ eq m^-3^ of bioreactor, a water footprint of 1.08 m^3^ m^-3^ of bioreactor, and an ozone depletion potential of 1.83 × 10^−5^ kg CFC11 eq m^-3^ of bioreactor, for an operation period of 5 days (Jacob-Lopes et al., 2020). In contrast, for a phototrophic production scenario using combustion gases as a carbon source and applying water recirculation, the reported values were 9.24 kg CO_2_ eq ash-free dry weight^-1^ for climate change, 8.02 m^3^ deprived ash-free dry weight^-1^ for water use, and 4.56 × 10^−7^ kg CFC11 eq ash-free dry weight^-1^ for ozone depletion (Lopes et al., 2023). It is important to note, however, that the comparability between life cycle assessment (LCA) studies is often limited by discrepancies in the definition of the functional unit and the delimitation of system boundaries. Hence, each case must be interpreted within its specific methodological context. Even so, when viewed from a broader perspective that considers productivity, scalability, and global applicability, heterotrophic cultivation emerges as a technically robust and increasingly relevant industrial alternative (Ruiz et al., 2022).

As an alternative to exclusively open or closed systems, hybrid photobioreactors have emerged as a promising strategy to balance process quality with economic feasibility. In this approach, the equipment integrates two or more distinct configurations, aiming to maximize the advantages of each (Deprá et al., 2019). For example, thin-layer systems can be coupled with bubble columns, in which parameters such as the height-to-diameter ratio are optimized to increase the surface-to-volume ratio. This configuration results in higher workload capacity, increased productivity, improved energy efficiency, and consequently, a relative reduction in costs (Padrón and Mérida, 2025).

Although they combine the best of both worlds—the operational simplicity of open systems and the microbiological safety of closed systems—hybrid photobioreactors still face significant challenges. While they can reduce costs compared to traditional closed configurations, such as tubular photobioreactors, their acquisition costs remain high compared to open systems. Studies indicate that the implementation cost of hybrid photobioreactors ranges from 3.91 to 15.08 kUSD/m^3^, depending on the quality of the materials used, whereas raceway ponds have much lower costs, in the range of 4–6 kUSD/m^3^. Tubular photobioreactors reach 40–50 kUSD/m^3^, and flat-panel photobioreactors can reach up to 150 kUSD/m^3^ (Ramírez-Mérida et al., 2017; Zhu et al., 2018). Thus, the high CAPEX of hybrid photobioreactors is directly related to material requirements and technological integration complexity, although it can be partially mitigated through scale-up and technological advances (Acién et al., 2012). In this context, techno-economic feasibility analyses and a clear definition of the application scale become crucial to guide strategic decisions and ensure rational adoption of these systems (Khor and Kang, 2025).

Furthermore, recent cost assessment studies highlight that downstream stages—such as concentration, harvesting, drying, and purification/storage—cannot be neglected, as they represent substantial fractions of total production costs (Vázquez-Romero et al., 2022; Kim et al., 2025). In particular, harvesting alone can account for 20%–30% of overall costs, while the combined costs of harvesting and extraction often exceed 50%–70% of OPEX, depending on the species cultivated and the technological route adopted (Tan et al., 2020; Geng et al., 2025). In this regard, strategies aimed at optimizing post-harvest operations become essential not only to reduce costs but also to strengthen the competitiveness of microalgae as accessible and sustainable food ingredients.

Therefore, the choice of cultivation system—whether open, closed, or hybrid—must be evaluated together with the demands of subsequent processing, as the goal is not only to maximize productivity during the cultivation phase but also to ensure efficiency and economic viability throughout the value chain. Establishing an appropriate trade-off between CAPEX, OPEX, and downstream requirements is fundamental to guiding microalgae production toward the food sector, transforming them into competitive raw materials aligned with safety, cost, and sustainability requirements (Korsah et al., 2025).

### 2.1 Integrated bioengineering approaches in microalgae cultivation

The efficiency and scalability of microalgae production depend directly on the choice and design of PBRs, which not only impose physical constraints on cellular growth but also define the main operational challenges that influence overall yields (Razzak et al., 2024). In this context, recent innovations have transformed the way these systems are conceived and operated. Technologies such as computational modeling, advanced automation, and Internet of Things (IoT) integration have expanded the ability to understand and control complex processes, including mass transfer, hydrodynamics, and heat dissipation, which were previously addressed in a fragmented manner (Thiviyanathan et al., 2022).

Computational modeling, for instance, enables the simulation of internal phenomena within PBRs and the anticipation of critical cultivation behaviors, guiding the development of more efficient geometries. Automation ensures that sensitive operations—such as nutrient dosing, medium circulation, and environmental monitoring—are carried out with precision and continuity, reducing the margin of human error. Modeling the relationships among fluid dynamics, light distribution, and growth kinetics in PBRs has contributed to improvements in reactor design and process control. This modeling performs photoperiod and mixing regime simulation through computational fluid dynamics (CFD) coupled with biological models (Gao et al., 2018; Papacek et al., 2018). IoT, in turn, connects smart sensors to real-time analytics platforms, allowing the dynamic adjustment of parameters such as temperature, pH, CO_2_ concentration, and light intensity, thereby converting the PBR into a responsive and intelligent system (Cui et al., 2020; Luzi and McHardy, 2022). Algae biotechnology is becoming increasingly innovative, integrating computational modeling and process control into production systems. Studies have demonstrate increased biomass productivity or process efficiency. In a study by Saini et al. (2021), a multi-objective genetic algorithm (CNNGA) hybrid algorithm was applied to optimize input parameters and increase phycobiliprotein levels and cell growth of the microalga Nostoc sp. The monitored input factors were BG-11, focusing on the components FAC, K2HPO4, MgSO4, and pH. The algorithm predicted optimal cultivation conditions, and thus an increase of 90% and 62% in biomass yield and phycobiliprotein recovery, respectively, was observed. In a study by Nayak et al. (2018), the optimization of environmental conditions in a Scenedesmus sp. was carried out using ANN-GA, and integrated with CO_2_ from combustion gases and domestic effluent. The biomass productivity (307.5 mg L^-1^ d^-1^) and lipid productivity (106.4 mg L^-1^ d^-1^) obtained in the optimized cultivation were 1.65 and 1.61 times higher, respectively, when compared with non-optimized cultivation.

This technological convergence inaugurates a new paradigm: rather than optimizing processes in isolation, it promotes an integrated approach that treats the PBR as a complete ecosystem. By aligning wet biomass control, energy management, and automated operation, it becomes possible to minimize economic bottlenecks, prevent contamination, detect deviations early, and continuously fine-tune cultivation. The result is greater productivity, consistency, and predictability in microalgae production systems (Gotovtsev et al., 2025).

However, the realization of this potential at an industrial scale still faces significant obstacles. The absence of standardized protocols, the limited long-term reliability of sensors, and the secure management of large volumes of data remain major barriers to full automation. Moreover, even with the support of digital tools, environmental and nutritional factors—such as temperature, pH, nitrogen and phosphorus balance, as well as the availability of inorganic and organic carbon—continue to be decisive for productivity, requiring continuous monitoring and increasingly sophisticated control strategies (Gil et al., 2025; Soon et al., 2025).

In this regard, although technological advances have improved operational predictability, microalgae cultivation remains vulnerable to environmental and biological fluctuations. This limitation highlights the need for complementary strategies, such as the selection of more adaptable and resilient strains, and the adoption of robust operational regimes (Borowitzka, 2013; Jebali et al., 2022). At this stage, microalgal biotechnology emerges as a strategic driver, integrating process engineering with biological solutions to maximize both productive efficiency and economic and environmental benefits.

This integration unfolds primarily along two convergent fronts: (i) genetic improvement of strains to enhance the yield of biomolecules of interest—such as proteins, fatty acids, carbohydrates, and pigments—through tools ranging from targeted mutagenesis to synthetic biology and metabolic optimization; and (ii) the application of these biological advances in scalable operational regimes, such as semicontinuous or continuous processes, with cultivation intensification through higher cell density and integration into existing industrial flows (Ahmad Kamal et al., 2024; Umetani et al., 2024).

To enable large-scale microalgae production, it is essential to combine biological enhancement of strains with advanced operational strategies. Contamination control, for instance, can be achieved through online monitoring, specialized reactor designs, or innovative genetic systems such as phosphite dehydrogenase–phosphite system (ptxD–Phi), which allows cultivation using phosphite (Dahlin and Guarnieri, 2022). In parallel, hydrodynamic management is crucial to minimize shear stress in sensitive cells, while computational fluid dynamics (CFD) modeling enables optimization of light distribution and mass transfer within reactors. These approaches must be assessed not only from a technical perspective but also in terms of cost and sustainability, ensuring that designed systems are feasible at an industrial scale (Borowitzka and Vonshak, 2017).

The selection of a cultivation system is equally strategic and depends on the characteristics of the species and the intended product. Open systems are generally used for more resilient species, such as *Arthrospira* spp., *Chlorella* spp., and *Dunaliella* spp., which can tolerate extremes of salinity or pH and reduce contamination pressure. For instance, *Dunaliella salina* thrives in saline environments, whereas *Arthrospira platensis* tolerates alkaline conditions (Qin et al., 2023). In contrast, PBRs allow for axenic cultivation and are preferred for high-value strains, such as *Haematococcus pluvialis* (astaxanthin), *Phaeodactylum tricornutum,* and *Nannochloropsis* spp (lipids), or *Euglena gracilis* (nutritional compounds), providing greater protection against contamination and the potential for high cell density (Penloglou et al., 2024).

These operational considerations lead to two complementary focal points in industrial application: first, the exploitation of market niches where the price per kilogram offsets higher production costs, such as pigments, nutraceuticals, and aquaculture ingredients; second, the integration and intensification of processes—including biorefineries, CO_2_ capture from industrial emissions, and the use of effluents as nutrients—to dilute costs and generate additional value through byproducts and ecosystem services (Leones-Cerpa et al., 2025). Technical strategies include hybrid processes (combining open systems with PBRs), operational density intensification, automation, and IoT for input optimization, and the development of value chains that internalize complementary services, such as water treatment and carbon capture (Deprá et al., 2019; Gotovtsev et al., 2025; Padrón and Mérida, 2025). Recent studies have systematically evaluated the effects of these strategies on biomass productivity and the yields of specific bioproducts across different microalgal strains, as summarized in Table 2.

**TABLE 2:** Applications of technological innovations and productivity gains in microalgae products.

Microalgae strain	Target bioproducts	Implemented strategies	Outcomes	References
*Haematococcus pluvialis*	Whole biomass and astaxanthin	Automated fed-batch PBRs with pH control (via real-time monitoring)	Biomass increased by +90.6% (up to 1.62 g/L) and astaxanthin productivity by 4.5 mg/L^-1^ d^-1^	Yang et al. (2021)
*Arthrospira platensis*	Whole biomass and CO_2_	Integrated hybrid system: open raceway + PBRs nested-bottled closed circuit	Biomass +38% (3.1 g/L), CO_2_ fixation +39.9% and photosynthetic efficiency +8.7%	Kubar et al. (2025)
*Limnospira fusiformis*	Whole biomass and phycocyanin	Automated mixers optimized by CFD + pulsed electric field (cellular stimulation)	Biomass productivity +20%; carbon fixation +43% (0.14 g/L^-1^ d^-1^); phycocyanin +14.4%	Ren et al. (2025)
*Nannochloropsis* sp	Whole biomass	Computational modeling of growth and scale simulations (ProviAPT)	Estimated biomass productivity up to 4.95 g/L^-1^ d^-1^ under optimized conditions	Ihadjadene et al. (2025)
*Chlorella saccharophila*	Lutein	Co-cultivation with symbiotic bacteria (optimized metabolism)	Lutein productivity of 298.97 µg/L^-1^ d^-1^ (+1.45× than monoculture); biomass +84%	Wu et al. (2025)
*Dunaliella salina*	Whole biomass and fatty acids	Red/blue monochromatic LED lighting (4:3 aspect ratio)	Biomass +10%; fatty acids +35%	Jin et al. (2021)
*Arthrospira maxima*	Whole biomass	Pulsed red/blue LED illumination (100–200 µs)	Biomass up to 3× greater than control with continuous light	Borella et al. (2022)

However, it is important to recognize that the majority of these investigations have been conducted at the laboratory scale. Translating these findings into industrial practice requires careful validation, as process performance and economic feasibility can differ significantly when scaled up. Consequently, pilot-scale validation of these strategies is essential. Detailed techno-economic assessments are required before full-scale implementation, as changes in cell characteristics—such as morphology or cell wall composition—can impact downstream processing costs, regulatory compliance, and genetic stability. Pilot testing and cost-benefit analyses are therefore prerequisites to ensure that gains observed in laboratory experiments are effectively translated into commercial competitiveness (Vázquez-Romero et al., 2022; Deprá et al., 2024).

Beyond cultivation, industrial viability depends on the integration of downstream processing. Harvesting, concentration, pretreatment, extraction, and purification of target biomolecules form a workflow that often determines the economic feasibility of the project. Among these stages, post-harvest drying is particularly critical due to its high energy consumption, which can render the final bioproduct cost-prohibitive in many scenarios. A promising alternative is direct extraction from wet biomass. This approach enables the use of aqueous-compatible techniques—such as solution-based extraction—and intensification technologies, including ultrasound, electric fields, and alternative solvents (Torres-Tiji et al., 2020). By reducing or eliminating the drying step, energy consumption is significantly decreased (Papachristou et al., 2023). Consequently, the decision to operate with dry or wet biomass ceases to be merely a procedural choice and instead dependent on reactor design, species selection, product objectives, and downstream efficiency, potentially converting productivity gains into tangible competitive advantages (Machado et al., 2025).

Overall, the success of industrial microalgae production relies on a holistic approach that integrates biological enhancement, reactor engineering, automation, operational control, and downstream strategies. While each innovation—from monitoring systems and IoT to biorefineries and wet biomass extraction—offers significant advancements, their isolated implementation does not guarantee efficiency or economic viability. True competitiveness emerges from the convergence of these elements, enabling the system to be simultaneously resilient, sustainable, and capable of producing high-value biomolecules. Nevertheless, the complexity of this technological ecosystem still demands rigorous testing, standardized protocols, and cost and sustainability analyses to ensure that productivity gains translate into tangible commercial outcomes.

## 3 Microalgae for the table: from ingredients to final products

Several microalgae species—such as *Arthrospira platensis, Arthrospira maxima, Isochrysis galbana, Nannochloropsis* sp.*, Tetraselmis* sp.*, Phaeodactylum tricornutum, Porphyridium cruentum, Picochlorum* sp.*, Chlorella vulgaris, Dunaliella salina,* and *Dunaliella tertiolecta*—have been recognized for their exceptional nutritional properties and high biotechnological potential (Occhipinti et al., 2024). Building on this inherent value, these species have established a strong presence in global markets, particularly in Asia, Europe, and North America, where their consumption has a long-standing tradition (Martínez-Ruiz et al., 2025).

Consequently, the discourse around microalgal biotechnology–today - extends beyond their nutritional composition to include societal perceptions and market dynamics, bringing microalgae into the spotlight. This surge in popularity reflects growing public interest in health-conscious, nutrient-rich diets and sustainable food systems. At the same time, food neophobia persists among some consumers, creating skepticism about the economic feasibility and tangible benefits of microalgae compared to traditional alternatives. The resulting tension between enthusiasm and hesitation underscores the challenges of translating promising laboratory findings into scalable, market-ready products. However, this very tension has spurred targeted research and product development, as companies and scientists strive to convert public attention into innovative functional foods (Schneider et al., 2023). In this way, the combination of rising consumer interest and active innovation has positioned microalgae as a focal point for advancing functional foods, opening new avenues for their broader adoption in global food systems (Bisht et al., 2024).

The diversity of microalgae species underpins the broad range of nutritional and functional properties that can be harnessed for food applications. Among these, *A*. *platensis* stands out for its highly digestible proteins with elevated biological value (460–630 g/kg of dry biomass), including essential amino acids such as leucine, isoleucine, valine, tryptophan, and methionine (Nikolova et al., 2024; Sinetova et al., 2024). Alongside *A*. *platensis*, *A*. *maxima* has drawn significant research interest. Both species are rich sources of polyunsaturated fatty acids (PUFAs), including γ-linolenic acid (18:3 ω-6), arachidonic acid (ARA, 20:4 ω-6), eicosapentaenoic acid (EPA, 20:5 ω-3), and docosahexaenoic acid (DHA, 22:6 ω-3), and their protein content can reach 600–710 g/kg of dry biomass (Shahidi and Ambigaipalan, 2018; Sinetova et al., 2024). *Chlorella vulgaris*, meanwhile, ranks among the most relevant species for nutritional applications due to its high protein content (up to 48% of dry weight) and phosphorus concentration (approximately 1761.5 mg/100 g of dry biomass). Its synthesis of β-1,3-glucan further supports immunostimulatory activity (Occhipinti et al., 2024). Additionally, *Chlorella vulgaris* provides a rich source of vitamins (A, B, C, and E) and minerals such as calcium, potassium, magnesium, and zinc, all of which are essential for metabolism, tissue regeneration, and DNA biosynthesis (Uchiyama-Tanaka et al., 2024; Wang et al., 2024).

In general, these microalgae species are cultivated conventionally, without any optimization of cultivation systems. However, when nutrient optimization processes or cultivation regimes are included, production values reach higher rates. For example, the optimization of continuous cultivation of the microalga A. platensis with the incorporation of urea (5 mmol L^-1^) showed a protein content of 1,295 mg L^-1^ of dry weight, 143% higher than control conditions (0.5 mmol L^-1^), where it showed an average protein content of 532 mg L^-1^ (Avila-Leon et al., 2012). Therefore, although the values of compounds extracted from microalgae in conventional systems are high, it is reasonable to consider that production in optimized systems further increases the levels of these compounds, mainly favoring commercial competitiveness.

Beyond their nutritional profile, the functional characteristics of microalgal components play a decisive role in their practical applications within food systems. These technofunctional properties govern how microalgae behave during preparation, processing, and storage, directly influencing texture, stability, and overall product performance (Caporgno and Mathys, 2018). Table 3 lists some of the technofunctional properties presented by microalgae in food. The microalgal proteins are beneficial in generating products with relevant functional properties, providing greater insight into agri-food chains. Solubility is an important factor in the application of proteins in the food industry, especially in emulsions and gels, as it directly contributes to other functional properties (Akbarbaglu et al., 2022). Microalgal-based proteins exhibit fascinating solubility, with a wide pH range. Furthermore, they demonstrate excellent emulsification, foaming, and gelation capabilities. Due to these protein attributes, microalgal-based proteins have a wide range of applications, including plant-based beverages, dairy analogues, baked goods, meat alternatives, snacks, and emerging formats, such as 3D-printed foods. Thus, when comparing the functional properties of microalgae-based proteins with those of plant-based proteins, such as rice bran protein, both are used in dairy desserts and demonstrate positive effects related to rheological and sensory characteristics. Furthermore, microalgae proteins promote clean production and mitigate the use of artificial emulsifiers and stabilizers (Prates, 2025).

**TABLE 3:** Technical-functional properties presented by microalgae-based compounds.

Microalgae	Compounds	Technical and functional properties	Mode of action
*Arthrospira platensis*	Protein	Oil/water absorption emulsifying foaming	Techno-functional enhancement with pH increased high oil absorption at a minor protein concentration. Foaming properties like egg proteins
*C. sorokiniana*	Protein	Gelling	Protein extracted formed heat-induced gels
*Chlorella vulgaris*	Protein	Emulsifying solubility	Enhanced emulsifying capacity properties of proteins. Solubilization of the protein extracted
*Dunaliella salina*	Microalgae biomass	Water absorption	Rheological properties of incorporated microalgae
*H. pluvialis*	Protein	Emulsifying	Strong potential of proteins isolated from H. pluvialis as an emulsifying agent
*Nannochloropsis oculata*	Protein	Solubility	Maximal solubility between pH 7 and 10
*Tetraselmis* sp.	Protein extracted	Solubility	Solubility is independent of suitable ionic strength and pH

Adapted from Pan-utai and Iamtham (2023).

These broad functional properties are directly related to the molecular and structural characteristics of microalgal proteins. In the case of *Chlorella* sp., it has been observed that immature cells of *Chlorella vulgaris* present only a single microfibrillar layer, whereas mature cells develop two layers, with a thicker outer wall. This structural difference influences protein extraction and bioavailability, thereby affecting properties such as solubility and the ability to form foams or emulsions. Moreover, the molecular weight of *Chlorella* proteins ranges from 14 to 116 kDa, depending on the culture conditions: lower molecular weight proteins (14–23 kDa, in autotrophic cultures) tend to display higher solubility and diffusion, favoring emulsifying and foaming properties, whereas higher molecular weight proteins (28–116 kDa, in photo-heterotrophic and mixotrophic cultures) contribute to greater viscosity and gelation potential. In addition, the richness in essential amino acids, such as histidine, isoleucine, leucine, lysine, methionine, phenylalanine, threonine, and valine, not only enhances nutritional value but also influences conformational stability and intermolecular interaction capacity, resulting in improved thermal stability and technological functionality (Costa et al., 2024; Wang et al., 2024).

The expression of these technofunctional properties in food systems, however, is not determined solely by protein composition or structure. Several process-related factors modulate their performance, including (i) biomass production, which defines the initial protein content and amino acid profile; (ii) pretreatment methods that can partially denature proteins; (iii) environmental parameters such as temperature, pH, and ionic strength; and, in some cases, (iv) purification steps, which may inadvertently reduce functional performance (Nunes et al., 2024). Effectively harnessing the full potential of microalgal proteins thus requires a holistic approach that considers both ingredient characteristics and processing conditions, highlighting the relevance of food design as a guiding framework.

Given this scenario, food design operates across all stages of the food production process—from cultivation to consumption and disposal—to overcome bottlenecks, spark innovation, and add value via a human-centered approach (Wolfert et al., 2023). Within this framework, the integration of living matter into products, structures, and processes naturally encompasses the use of microalgae (Vrenna et al., 2021). Their incorporation into foods, however, does not occur as a complete replacement of conventional ingredients; to date, there are no widely commercialized products in which microalgae serve as the sole base ingredient. Instead, they are typically added in a complementary manner, whether through partial substitution of wheat flour in breads and pastas, as a protein fraction in plant-based burgers, or in the form of extracts aimed at delivering specific functionalities. Beyond these conventional approaches, advanced technologies such as nano- and microencapsulation, as well as 3D food printing, have expanded the possibilities for integrating microalgal biocompounds, ensuring stability, functionality, and consumer acceptance (Sukhikh et al., 2022; Caporgno and Mathys, 2018; Verni et al., 2023).

Microalgae-derived biocompounds, when incorporated directly into food matrices, face several technical challenges, including low solubility, susceptibility to chemical degradation, undesirable flavor profiles, limited bioavailability, and reduced bioactivity (Vieira et al., 2020a). To overcome these limitations, nano- and microencapsulation have emerged as a promising strategy. Technically, encapsulation involves the entrapment or coating of an active agent within a carrier material, forming a particulate system that can accommodate substances in solid, liquid, or gaseous forms. Nanoparticles range from 1 to 100 nm, while microparticles span 100 nm to 1,000 μm, providing flexibility in design and functionality (McClements, 2018). In this context, when applied to microalgae-based foods, the encapsulation has demonstrated clear advantages. For instance, astaxanthin delivered via lipid-based carriers exhibits enhanced antioxidant activity and greater stability during processing, while lutein-loaded nanocapsules show superior bioavailability compared to their free form (Pan et al., 2024; Sorasitthiyanukarn et al., 2022). Encapsulation efficiencies (EE) for microalgae-based bioactives, such as astaxanthin, phycocyanin, and phenolic extracts, vary between 40% and 90%, depending mainly on the wall material used and the encapsulation technique (Vieira et al., 2020a; Martínez-Álvarez et al., 2020; Machado et al., 2022). Studies on controlled release follow the Korsmeyer-Peppas model, based on diffusion, with adjusted release profiles (Londoño-Moreno et al., 2023). Thus, the release occurs over a period of 4–24 h, resulting from the gradual diffusion of hydrophilic pigments and antioxidants under simulated gastrointestinal conditions (Martínez-Álvarez et al., 2020; Panagiotakopoulos and Nasopoulou, 2024). In a study by Pan et al. (2024), when astaxanthin was encapsulated in liposomes, its bioavailability increased to ∼40%, compared to the bioavailability of free astaxanthin (∼6.5%). Furthermore, when analyzing sodium alginate coatings, the bioavailability of astaxanthin increased by approximately 70% after the alginate was exposed to an environment with a pH close to neutral in the intestinal digestive solution (Huang et al., 2024). These examples illustrate how encapsulation can transform compounds that are otherwise limited in application into viable functional food ingredients. Nevertheless, despite these promising outcomes, encapsulation itself faces practical constraints. High production costs, uncertainties regarding scale-up, and regulatory challenges continue to hinder its widespread industrial adoption (Tarhan, 2020; Vitale et al., 2025). Consequently, while encapsulation represents a cutting-edge approach with significant potential, its full realization in the food sector will require integrated advances in food nanotechnology, cost-reduction strategies, and clear regulatory frameworks.

Related advances in efficient strategies involving fermentation approaches and enzymatic treatments can be the basis for making microalgae an ingredient of interest in breads, yogurts, and other fermented foods. Incorporating microalgae as an ingredient in formulations increases protein content, provides peptides and bioactive pigments, and improves water retention and texture properties in bakery and dairy matrices (Verni et al., 2023; Mosibo et al., 2024). However, persistent bottlenecks in the inclusion of whole biomass include strong, unpleasant flavors, dense cell walls that hinder nutrient bioaccessibility, and color changes (Olsen et al., 2024). As a result, enzymatic hydrolysis—through proteases and carbohydrases—as well as controlled fermentation processes—with lactic acid bacteria or yeast—provide the rupture of these rigid cell walls, resulting in the release of soluble peptides and nutrients, minimizing the levels of undesirable volatiles while simultaneously forming desirable aroma precursors. This results in improved digestibility, functionality, and sensory profiles (Parsaeimehr and Ozbay, 2024; Garofalo et al., 2022). Therefore, fermentation techniques and enzymatic treatments can transform microalgae into a favorable ingredient for inclusion in breads and yogurts.

**FIGURE 5:**
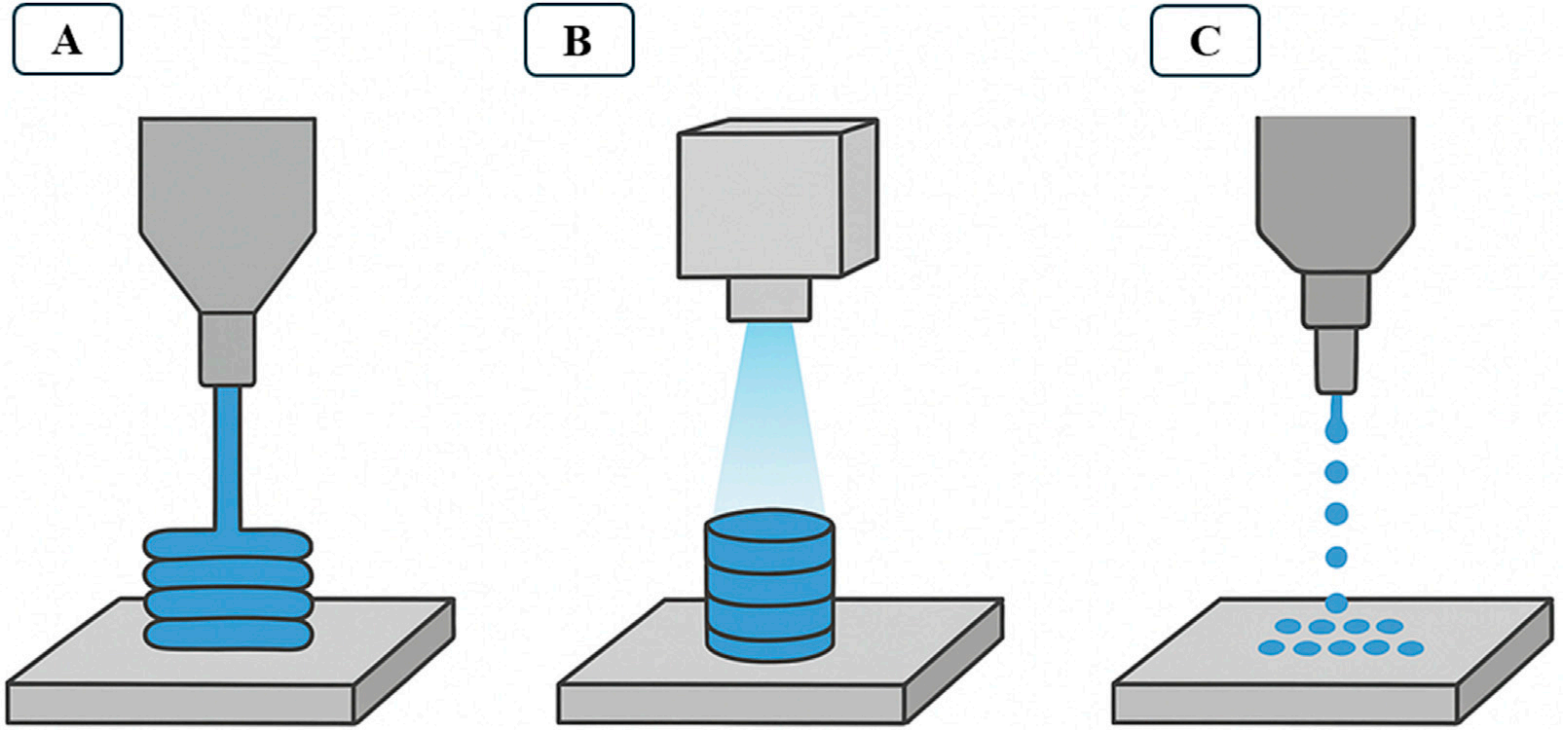
Schematic representation of the three 3D bioprinting systems: **(A)** Extrusion-based; **(B)** Light-projection-based; **(C)** Inkjet-based.

On the other hand, 3D food printing is also on the rise, emerging as a disruptive technology in food design. Unlike nano- and microencapsulation, this technique focuses on producing complex and personalized geometric structures, combining nutrition with multiple flavors, colors, and textures. But the 3D printing boom goes further, also offering economic and environmental benefits, reducing waste and enhancing underutilized ingredients (Vieira et al., 2020b). One example of a current application is the incorporation of microalgae-based biomass, which has great nutritional appeal but still has a high rejection rate due to its individual characteristics, such as color, flavor, and intense odor (Letras et al., 2022). Thus, by incorporating microalgae into multisensory food printing, these negative aspects are masked, and consequently, market acceptance is increased. There are essentially three approaches to bioprinting: extrusion-based systems, light-projection-based systems, and inkjet-based systems, as illustrated in Figure 3. Extrusion-based systems, the most widely used for microalgae, operate with high-viscosity bioinks containing living cells. After the bioink is prepared, it is mechanically extruded through the printing nozzle onto the printing platform (Figure 5A). Light-projection-based systems operate with photosensitive materials (Figure 5B). During printing, the materials are directly exposed to UV light patterns for layer-by-layer polymerization, resulting in higher print resolution. Finally, inkjet-based systems do not involve direct contact (Figure 5C); bioink droplets are applied to predefined locations with the aid of piezoelectric or thermal actuators (Tang et al., 2025). Each of these systems has advantages and disadvantages that affect the viability of the application in the food industry.

While there is great promise, there are visible bottlenecks. The high cost and scalability of bioinks, consumer acceptance of printed foods, and regulatory needs due to safety and labeling still pose an unresolved challenge (Guaqueta-Garcia et al., 2025). Therefore, even if there is the illusion that the microalgae market can be transformed from a supplement source to a conventional ingredient, success will only be achieved when there is a consensus between technology, accessibility, trust, and safety.

From the perspective of food processing, therefore, the successful inclusion of microalgae in conventional diets will depend largely on strategies capable of mitigating their sensory and technological limitations. Approaches such as micro- and nanoencapsulation can mask undesirable flavors and aromas, improve stability, and enhance nutrient bioavailability, while partial incorporation into flour-based matrices enables nutritional enrichment without compromising consumer acceptance. Fermentation and enzymatic treatments further contribute to reducing off-flavors and increasing digestibility, whereas the use of matrices with strong or complex sensory profiles—such as sauces, snacks, or fermented products—can naturally mask their characteristic taste and color. Emerging technologies, including 3D food printing and high-pressure processing, provide additional opportunities to optimize texture, design, and functional properties, making microalgae more appealing to consumers. Ultimately, these integrated processing strategies allow microalgae to transition from a niche supplement to a functional ingredient embedded in attractive, sustainable, and culturally acceptable food products, closing the gap between their nutritional potential and real-world dietary integration.

## 4 Algae on the menu: understanding how consumers embrace novel food design

The advance of microalgae biotechnology, allied to the most recent scientific discoveries, offers great potential for food innovation, but faces a central challenge: understanding the factors that really influence consumer oil. The success of microalgae-based products on the market depends on the perception and decisions of consumers, making it essential to investigate the elements that shape their choices and preferences in relation to these foods—factors that, at this time, make it difficult for microalgae to find their place on the daily table of consumers.

Beyond consumer perception, the microalgae-based food sector is experiencing growth driven by the alternative protein market, with projections reaching USD 1,298.25 million by 2032, exhibiting a compound annual growth rate (CAGR) of 8.61% (Fortune Business Insights, 2025). This increasing demand for sustainable protein sources, natural colorants, and functional ingredients directly reflects the growth of the microalgae-based market. Thus, recognizing microalgae as a high-value raw material for the food, nutraceutical, and beverage industries highlights its market potential, particularly for applications such as plant-based meat analogues, fortified dairy alternatives, and functional beverages (Saeed et al., 2025).

In this context, sensory experience emerges as one of the main determining factors for the acceptance of microalgae-based foods. Despite its recognized nutritional value, the addition of these biomolecules can cause perceptual alterations in color, taste, and flavor, which frequently generate resistance on the part of consumers (Barros de Medeiros et al., 2022; Verni et al., 2023; Nunes et al., 2024; Olsen et al., 2024). This skepticism is associated, above all, with negative perceptions related to the palate. Studies conducted by Michel et al. (2021) demonstrate that, when compared to traditional beef burgers, those formulated with microalgae-based proteins arouse significantly lower sensory expectations. Evidence such as this reinforces that attributes such as appearance, texture, taste, and flavor play a decisive role in the acceptance of new food design (Appiani et al., 2023).

However, a question arises as to whether these sensory limitations are intrinsic to the biochemical composition of microalgae—such as chlorophylls, carotenoids, phenolic compounds, and volatile metabolites that form earthy, marine, or sulfurous notes—or whether they are mostly a consequence of cultural and perceptual issues. However, some studies indicate that both aspects interact dynamically. On the one hand, volatile compounds such as dimethyl sulfide, hexanal, and geosmin are associated with the “undesirable flavors” present in microalgae biomass (Kumar and Sharma, 2023; Wang et al., 2024; Wu T. et al., 2022). On the other hand, consumer expectations and cultural familiarity with traditional flavors influence perception. In other words, it is assumed that consumers accustomed to plant-based or fermented products show a greater tolerance to these taste notes, which may suggest that cultural exposure mitigates rejection (Zielińska et al., 2025; Prates, 2025). Thus, sensory gaps are not only attributed to intrinsic biochemical properties but are also linked to consumers’ sensory interpretation in a psychological and symbolic way.

This question becomes even more critical in the functional food segment, considered one of the main industrial niches for the application of microalgae. These products, generally marketed at higher prices, consumers tend to demand that sensory excellence be at the level of the value paid, becoming a central selection criterion (Ponte et al., 2025).

In addition to sensory barriers, the lack of knowledge about microalgae constitutes another significant obstacle to their widespread acceptance. As a still relatively unfamiliar ingredient, many consumers tend to prioritize foods that provide immediate and tangible benefits, while resisting changes that require adaptations in taste. This behavior partly reflects the predominance of a “child-like palate” in contemporary food choices, where simple, sweet, or culturally established flavors are preferred over new or more complex sensory profiles. Consequently, it becomes particularly challenging to convince consumers to forgo established attributes—especially taste—even when socially desirable advantages, such as greater sustainability, are present (Lafarga et al., 2021; Maehle and Skjeret, 2022).

However, to overcome biochemical and cultural barriers, a set of strategies combining formulation and marketing is necessary. From a technological perspective, reducing undesirable odors and flavors to preserve nutritional integrity is achieved through the encapsulation of pigments and volatile compounds, the fractionation of proteins, and enzymatic treatments (Verni et al., 2023; Wang et al., 2024). From a perception perspective, reshaping the idea of strangeness and sensory doubt into a positive attribute is accomplished through narrative marketing that highlights sustainability and functionality. Therefore, culinary workshops and collaborations with chefs can be effective ways to reframe consumer expectations, while creating positive sensory experiences (Van der Stricht et al., 2024; Li et al., 2025). In this way, these approaches can help overcome the biochemical and perceptual barriers to accepting foods containing microalgae.

In this context, studies suggest that everyday foods, such as bakery products and fermented alcoholic beverages like beer, may serve as strategic vehicles to introduce microalgae into the diet, particularly attracting environmentally conscious and innovation-oriented consumers, although this still represents a developing niche (Maehle and Skjeret, 2022). Similar findings were reported by Grahl et al. (2020) when evaluating filled pasta formulated with different concentrations of *Arthrospira* sp.-soy extrudate. The flavor variations of lemon-basil, tomato, and beetroot-ginger showed that, although the lemon-basil combination better masked the characteristic earthy and musty notes of the microalgae, acceptance decreased significantly as the concentration of the ingredient increased. Overall, consumers exhibited greater receptivity to formulations resembling traditional foods, suggesting that familiarity plays a central role in the acceptance process.

However, just like sensory and cultural aspects, the microalgae-based food industry also faces structural and economic challenges that hinder its large-scale consolidation. Some of these challenges include high production costs in the cultivation and processing stages, the limited scalability of cultivation systems such as photobioreactors, the variability in biomass composition that largely depends on the strain and cultivation conditions, and the need for the implementation of harmonized regulatory frameworks across regions (Jacob-Lopes and Zepka, 2023; Kathuria et al., 2024). Furthermore, the combination of consumer education and product distribution logistics remains underdeveloped, resulting in significant bottlenecks in the commercialization stages. Strengthening these areas is fundamental, especially to overcome the gap between scientific innovation and market insertation.

As a strategy to expand consumer acceptance, companies in the microalgae-based food sector are investing in innovations that reconcile nutritional benefits with more attractive sensory profiles. A recent example is Aliga Microalgae, which has advanced the development of white *Chlorella*, a chlorophyll-free strain with a neutral flavor and white color, opening new formulation possibilities previously unavailable (Aliga, 2025). Similar initiatives have already been led by companies such as Algenuity and Allmicroalgae. In 2019, Algenuity launched additives derived from *Chlorella vulgaris* with significantly reduced chlorophyll content, while Allmicroalgae began to produce biomass of the same species with enhanced sensory profiles. Both companies use heterotrophic cultivation—by fermentation with glycose as a carbon source—to obtain food-quality biomass, achieving shades that vary between yellow, lime-green, and white. These advances demonstrate the persistence of the sector in overcoming sensory barriers, making microalgae-based products more versatile and promising for the acceptance on the market.

In light of these innovations, it becomes essential to understand not only the technological potential of microalgae but also how consumers respond to such changes. In this context, scientific investigations play a central role in assessing the acceptance of products formulated with new variants, such as white *Chlorella*. A study conducted by Cabrol et al. (2023) demonstrated that the inclusion of this microalga in frankfurter-type sausages enriched the nutritional profile of the product, increasing protein content, dietary fiber, minerals (calcium, potassium, phosphorus, and zinc), and polyunsaturated fatty acids (PUFAs), without significantly compromising its technological quality. The most noteworthy finding, however, was sensory: even with subtle changes in color and texture, the samples received favorable acceptance from 20 panel participants.

Despite these advances, a large portion of microalgal biomass applications still face consumer resistance. Studies have explored its use in pasta and bread (Qazi et al., 2022), couscous (Khemiri et al., 2021), snacks (Uribe-Wandurraga et al., 2020), cookies (Prashant Sahni and Shere, 2018), fish burgers (Atitallah et al., 2019), turkey burgers (Marti-Quijal et al., 2019), pork liver pâté (Zamuz et al., 2019), chicken rotti (Parniakov et al., 2018), and dairy products (de Oliveira and Bragotto, 2022; Hernández et al., 2022). However, many of these products, when subjected to tasting, are evaluated negatively, mainly due to their characteristic color, aroma, and flavor. In this context, the advances achieved with the production of white *Chlorella* may represent the “escape valve” the industry has been waiting for to overcome sensory barriers and finally secure a stable presence on supermarket shelves. In a way, the available literature already allows for a clearer understanding of consumer psychology when it comes to microalgae-enriched foods.

Given this scenario, it becomes clear that, to incorporate microalgae-based foods into consumers’ diets, sensory improvement alone is not enough; it is also necessary to strengthen communication and marketing strategies that support this goal. Education on healthy and renewable food ingredients, clear and attractive labeling, and the application of established marketing techniques (stimulating curiosity) can build trust among consumers of alternative foods (Wassmann et al., 2024; Li et al., 2025). Targeting products to specific consumer niches, such as individuals committed to sustainable eating, athletes, and early adopters of innovation, further increases the likelihood of success. Moreover, nutritional re-education for young adults may provide a potential pathway for the introduction of microalgae-based products. This approach, combined with transparency regarding all nutritional and environmental benefits, can help reduce negative perceptions (Van der Stricht et al., 2024; Wassmann et al., 2024). Currently, one strategy that has proven effective for many products—even those initially perceived as “challenging” — is adoption by chefs and culinary influencers, who act as cultural and sensory mediators between the product and the consumer. So, why not apply this approach to foods that can truly transform the diet? By incorporating microalgae into gourmet dishes, a positive sensory experience—in terms of taste, texture, and presentation—is created, helping to shape cultural acceptance of the ingredient, reduce prejudices, and enhance its perceived value (Li et al., 2025).

From a critical and integrated perspective, the trajectory of microalgae in the food sector can be understood through three strategic pillars, which, although interdependent, have distinct impacts on their consolidation. At the core of this evolution lies regulation and the scientific validation of functional and health benefits: without formal approval, any technological advancement or marketing appeal remains limited, as consumer trust and food safety are non-negotiable prerequisites for entry into established markets. In parallel, continuous research and development play a critical role, not only in enhancing nutritional potential but also in refining sensory attributes—taste, aroma, texture, and appearance—that determine whether a product will be effectively accepted. Alongside this, education, communication, and marketing function as strategic tools to translate science into perceived value, build credibility, and encourage gradual adoption, particularly among innovative or environmentally conscious consumer niches. Finally, while the sustainability narrative remains relevant, it should be regarded as a reinforcement to positioning rather than an isolated factor in purchasing decisions. Only when these elements—regulation, scientific evidence, sensory innovation, and strategic communication—act in an integrated manner do microalgae move beyond a technological promise to fulfill their true role: microalgae on the menu, incorporated in a tangible, reliable, and appealing way into the global diet.

## 5 Ensuring safety of microalgae-based products: regulatory guidelines and evaluation methodologies

Microalgae offer a unique combination of nutritional and environmental attributes, making them promising candidates as innovative food ingredients. However, the insertion of these microorganisms into the food chain requires rigorous safety validation criteria, since the simple presence of nutritional value or bioactive compounds does not ensure safety. In this context, three pillars converge in international assessment systems: the absence of toxicological effects, the stability of the nutritional composition, and the confirmation of historical consumer assurance. Thus, each country or economic block structures its processes differently, reflecting not only legal differences but also historical and cultural aspects of its relationship with innovative foods.

In the European context, for example, innovative food ingredients, including microalgae, are classified as novel foods (EU Regulation 2015/2283) (EFSA Panel on Nutrition et al., 2022). Before being marketed, these products must undergo prior evaluation by the European Food Safety Authority (EFSA), which carries out a multidimensional analysis, considering potential adverse effects, nutritional composition, consumption history, and toxicity (Cruz and Vasconcelos, 2023). In the United States, meanwhile, the safety endorsement follows a specific approach called GRAS (Generally Recognized As Safe) issued by the Food and Drug Administration (FDA). This regulatory pathway recognizes substances as safe for human consumption based on scientific evidence, including acceptance and a documented history of safe use. Unlike the formal European process, the GRAS status allows certain ingredients to be used without individual submission to the agency, the producer or manufacturer remains responsible for ensuring product safety. Such a model confers greater market agility, but also transfers greater responsibility to the manufacturer regarding safety assurance, compared to the preventive and centralized nature of the European system. These differences in approach are not merely procedural: they directly influence the speed of innovation and consumer confidence. While the European model privileges a logic of caution, reducing regulatory risks, the North American system favors greater flexibility, but raises criticism about potential control gaps. In both cases, the legitimacy of microalgae as food ingredients depends on robust analyzes that reconcile scientific evidence and legal requirements.

In terms of the practical application of these regulations, various microalgae may be recognized by international safety standards. In the European Union, species such as *Arthrospira* sp., *Chlorella* sp., *Porphyridium cruentum,* and *Crypthecodinium cohnii* are validated and have GRAS status or equivalent, while another six species–including *Haematococcus pluvialis*, *Phaeodactylum tricornutum*, *Dunaliella* sp., *Nannochloropsis* sp., *Nitzschia* sp., and *Schizochytrium* sp. – are considered safe, without reports of toxins contained (Jacob-Lopes and Zepka, 2023). In the United States, the FDA approved various microalgae for human consumption, highlighting *Arthrospira platensis*, *Chlorella vulgaris*, *Haematococcus pluvialis*, and *Tetraselmis chuii*, recognized for their high nutritional value and potential as sustainable sources of proteins, polyunsaturated fatty acids (PUFAs), and antioxidants (Andrade-Bustamante et al., 2025).

**FIGURE 6:**
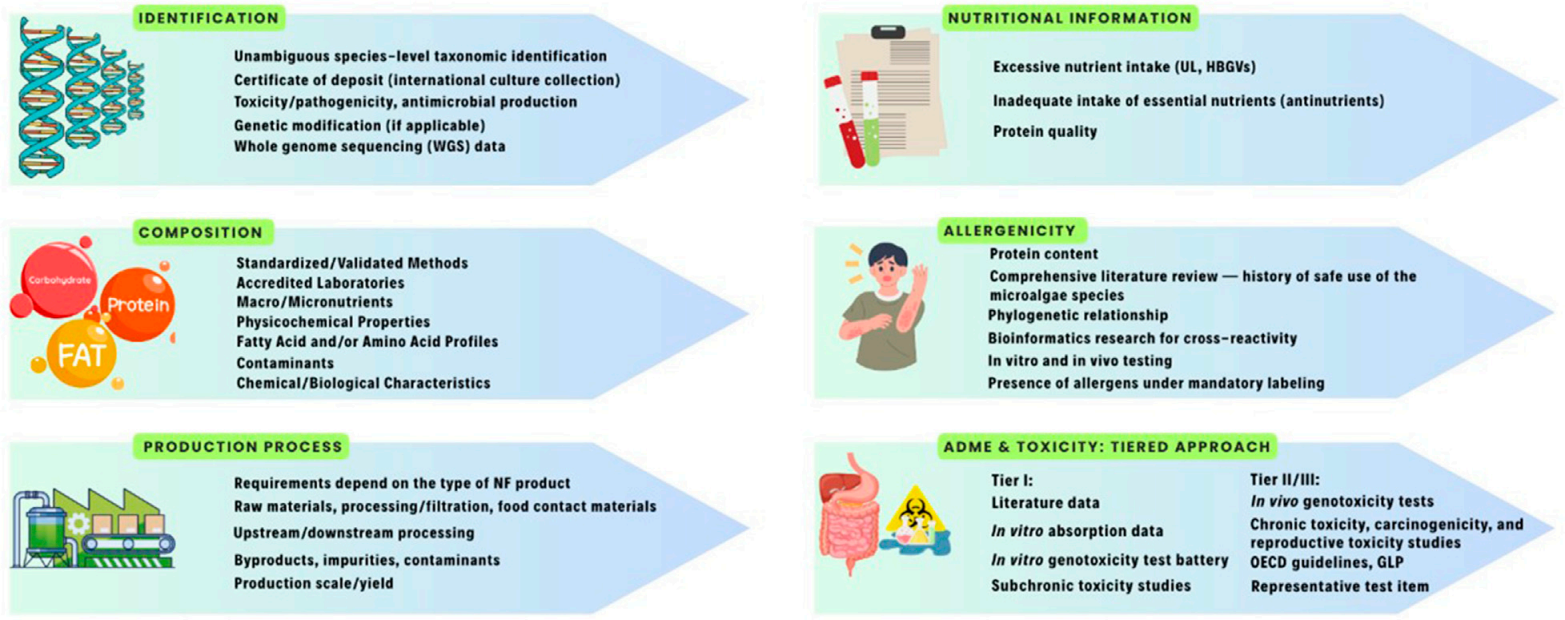
Key scientific criteria employed by EFSA for evaluating the safety of microalgae-based innovative foods. NF: Novel Food; UL: Upper Levels; HBGV: Health-Based Guidance Values; GLP: Good Laboratory Practices.

Although several microalgae species already hold formal safety recognition, as previously described, the regulatory landscape remains dynamic and constantly evolving. In 2024, for instance, EFSA issued around eleven opinions on novel foods derived from microalgae, while six additional evaluations are still ongoing (Garciarena et al., 2025). This demonstrates that, even with well-established nutritional and functional advantages, the safe development and consumption of microalgae-based products rely on meeting rigorous technical criteria that ensure food safety and consumer protection. Figure 6 illustrates the main parameters EFSA uses to assess the safety of novel foods derived from microalgae.

As observed in Figure 6, the safety assessment of microalgae-based products involves a series of interdependent parameters, ranging from strain identification to detailed analysis of composition and the absence of contaminants. The starting point is taxonomic identification, which ensures that the species used corresponds exactly to the one approved for consumption. In this context, biotechnology plays a central role, enabling genetic validation through sequencing techniques such as 18S/25S rRNA or whole genome sequencing (WGS). Furthermore, the strain under evaluation must have a valid deposit certificate issued by an internationally recognized culture collection, ensuring traceability and reliability (Garciarena et al., 2025).

The second essential aspect concerns the production process of novel foods (NFs), which must comply with rigorous food safety standards. This includes adherence to good manufacturing practices (GMP), the implementation of hazard analysis and critical control points (HACCP), and alignment with international standards such as ISO 22000 (Food Safety Management System) and Regulation (EC) No. 852/2004 on food hygiene (Zanatta et al., 2023; Kathuria et al., 2024; Precup et al., 2024). During the microalgal biomass production, there are potential risks of environmental contamination by chemical agents (pesticides, heavy metals, herbicides), biological hazards (bacteria, fungi, cyanotoxins), and physical contaminants (microplastics), making continuous monitoring indispensable (Ferreira de Oliveira and Bragotto, 2022; Wu G. et al., 2022; Mendonça et al., 2024).

Compositional characterization, in turn, constitutes one of the central pillars of safety assessment. This stage involves a detailed analysis of macro- and micronutrients, pigments, polysaccharides, and other compounds with bioactive activity (de la Noue and de Pauw, 1988; Ampofo and Abbey, 2022; Davani et al., 2022; Ferreira de Oliveira and Bragotto, 2022; Ruzik, 2023), as well as the detection of contaminants originating from cultivation or processing, including mycotoxins, polycyclic aromatic hydrocarbons, residual solvents, and dioxins (Kumar and Sharma, 2021; Salehipour-Bavarsad et al., 2024; Garciarena et al., 2025). At this stage, validation of the declared nutritional profile is also performed, ensuring that levels of proteins, essential fatty acids, vitamins, and minerals effectively correspond to the reported composition (Salehipour-Bavarsad et al., 2024; Garciarena et al., 2025).

**FIGURE 7:**
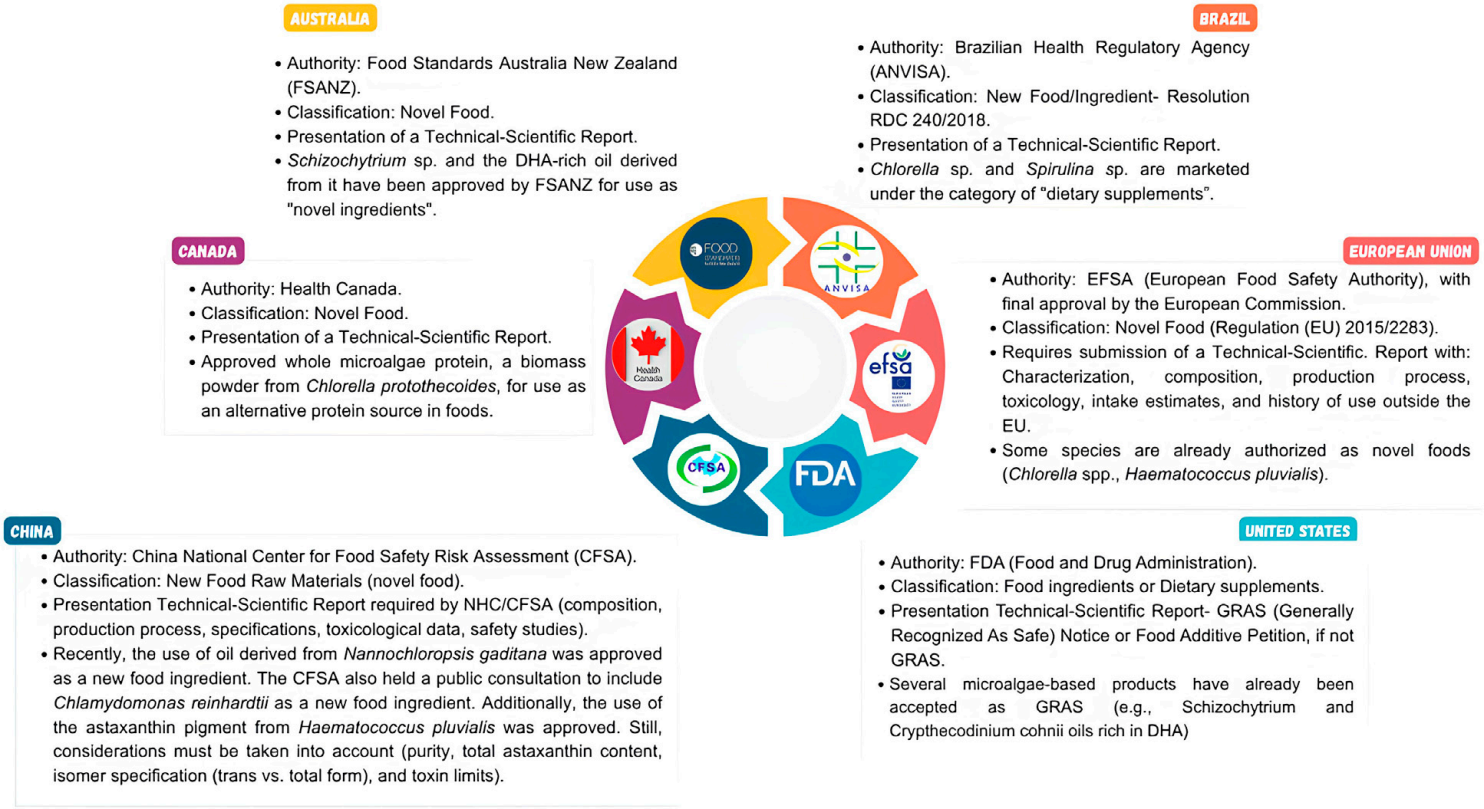
Regulatory pathways for novel microalgae-based foods in different regions of the world.

Despite a common foundation, there is considerable global regulatory diversity. In Brazil, for instance, the Brazilian Health Regulatory Agency (ANVISA) requires that new food ingredients undergo rigorous toxicological and nutritional assessments before commercialization, following the framework established for “novel foods” (Health Regulatory Agency, 2023). In China, the National Health Commission (NHC) regulates such products under the system of “New Food Raw Materials,” in which each microalgal species must be individually approved with the submission of detailed data on composition and safety (Ettinger and Li, 2025). In other countries, the approach is also specific. Australia and New Zealand have adopted a joint system through Food Standards Australia New Zealand (FSANZ), in which novel food applications are assessed on a case-by-case basis, focusing on dietary exposure and allergenic risks (Kedar et al., 2024). In Canada, Health Canada oversees the approval process through its “Novel Foods” regulations, emphasizing substantial equivalence and consumer safety (Health Canada, 2022). Despite differences in terminology, specific criteria, and scope of analysis, these systems share a common evaluation framework: all require toxicological testing, detailed compositional characterization, and historical documentation of safe consumption. Figure 7 illustrates how regulatory pathways for microalgae-based novel foods are structured in Brazil, China, Australia, and Canada, highlighting both the diversity and the points of convergence among international approaches.

Although regulatory systems require precise and detailed data on the composition and safety of microalgae, biomass characterization still faces methodological challenges that may compromise interpretation and regulatory validation processes. Most commercial methods currently employed were not originally designed for microalgae, which can affect the accuracy and comparability of results. In addition, methodological inconsistencies across laboratories hinder data interpretation, while significant redundancies in method selection—such as in lipid determination—highlight the need for harmonization and standardization. Overcoming these gaps is therefore essential to meet regulatory criteria and ensure the safety and quality of microalgae-based products.

Recent studies provide concrete evidence of these methodological limitations. Lane et al. (2021), for instance, observed that the analytical methods currently in use, largely derived from conventional food protocols, were not specifically developed for microalgae. In their research, they found that laboratories employed different methods, leading to minor inconsistencies: when analyzing the same strain biomass, protein and ash values were consistent only when laboratories followed identical protocols. However, lipid determination showed significant variation depending on the protocol adopted, which also affected carbohydrate estimates. Similarly, Cheah and Chan (2022) emphasize that methodological divergence persists due to the lack of consensus among researchers on which techniques to adopt, complicating the comparison and interpretation of results. In this scenario, standardization of methodologies, establishment of consistent protocols, and development of analytical techniques tailored to microalgae emerge as essential solutions (Morshchinin, 2025). The absence of standardized analytical methods underscores the need for biotechnology, already advanced in certain areas, to concentrate efforts on the early stages of the process, thereby ensuring the safety and quality of microalgae-based products.

Given that several microalgae species are already widely used for human consumption, current research seeks to improve the assessment of absorption, distribution, metabolism, and excretion (ADME) processes—an essential aspect for the introduction of novel bioactive compounds or chemically modified fractions. Zuorro et al. (2024) emphasize in their study the need for preclinical trials evaluating the ADME of microalgae-derived bioactive compounds for antimicrobial applications. Furthermore, EFSA Panel on Nutrition et al. (2023) details the pharmacokinetics (ADME) of fucoxanthin and its metabolites in the microalga *Phaeodactylum tricornutum*. To predict the absorption and distribution of bioactive peptides from *Nannochloropsis gaditana*, Paterson et al. (2025) performed an *in silico* assay. In addition, ADME analysis of bioactive microalgal compounds with pharmacological potential has been conducted through computational approaches (Prasetiya et al., 2023). Biotechnology is crucial in these stages, as it facilitates toxicological evaluations through *in vitro* and *in vivo* assays. Beyond improving allergen risk identification, it provides essential data for the establishing safe intake limits and specific labeling of products intended for human consumption (Salehipour-Bavarsad et al., 2024).

Finally, the existence of specific regulations across different regions of the world reinforces the notion that the traditional diet is no longer the sole protagonist on the consumer’s plate. Microalgae, once properly assessed and regulated, are ready to take their place on supermarket shelves. Although certain advances are still required, the path ahead remains promising: considering that consumers have already accepted larvae and insects as ingredient bases in various foods, there is considerable potential for microalgae to secure their position as well.

## 6 An outlook on the sustainability of microalgae-based foods

The growing interest in including microalgae in food formulations is attributed not only to their high nutritional value and broad bioactive activity, but also to their distinctive metabolic pathways, which rely predominantly on the capture of carbon dioxide (CO_2_) as the basis for nutrient assimilation. This metabolic feature allows microalgae to contribute simultaneously to pollutant removal and, in certain contexts, to alleviating the demand for arable land when compared with conventional agriculture, reinforcing their role as an ecologically viable resource (Abdelfattah et al., 2023; Diaz et al., 2023; Chen et al., 2024; Iñiguez-Moreno et al., 2025).

Furthermore, its application in food design can be related to several Sustainable Development Goals (SDGs) of the United Nations (UN) 2030 Agenda, including zero hunger (SDG 2) through combating malnutrition, health and wellbeing (SDG 3) via bioactive compounds that promote health, responsible consumption and production (SDG 12) by requiring less land and water while offering high protein yield per area, climate action (SDG 13) through sequestering CO_2_ and replacing conventional proteins, and life below water (SDG 14) by reducing pressure on fish stocks (Mariutti et al., 2021; Iñiguez-Moreno et al., 2025). However, it is necessary to carry out a critical analysis regarding the sustainability of its production, considering the three dimensions of the triple bottom line: environmental, economic, and social. Although the discourse surrounding microalgae production promotes it as an environmentally viable innovation, its practice is closer to a utopian projection than a robust reality.

From an environmental perspective, the narrative surrounding microalgae is about their major impact on bioremediation, where they capture CO_2_ from the atmosphere and absorb pollutants from wastewater, while also producing value-added biomass (Bhatt et al., 2022; El-Sheekh et al., 2025). Studies show that microalgae cultivation can fix between 1.83 and 1.88 kg of CO_2_ per kilogram of dry biomass produced, contributing significantly to the net reduction of greenhouse gas release when associated with industrial carbon sources (You et al., 2022; Picknell et al., 2025). Similarly, using wastewater can also increase the productivity of compounds while decreasing dependence on external nutrients. According to You et al. (2022), the treatment of wastewater from palm oil mills using the microalgae *C. pyrenoidosa* can achieve a lipid production of 68% and nutrient removal of 71%.

However, to achieve satisfactory productivity of high-purity biomass, water quality must be high, ensuring ideal growth conditions and preventing contamination. In this case, freshwater resources are generally used, which can lead to competition with other sectors and exacerbate water scarcity. Although the use of wastewater as an alternative source for microalgae cultivation is possible, high levels of pollutants, such as pesticides and heavy metals, can cause physiological stress and, consequently, reduce yield, beyond the risk of bioaccumulation of toxic compounds that can compromise the quality of the algal raw material. This problem, however, can be solved by employing advanced technologies and treatment processes, but these tools require high costs and large amounts of energy (Su et al., 2023; de Paula Pereira et al., 2024; Ugya et al., 2025).

Similarly, closed cultivation systems, such as photobioreactors, while providing optimal growth conditions with controlled abiotic factors such as light, temperature, and nutrient levels, require high electrical demand (Rehman et al., 2022). This affects the economic viability of the process, in addition to potentially increasing environmental impacts due to indirect carbon emissions, especially if the energy used comes from non-renewable energy sources (Ugya et al., 2025). According to Dias et al. (2022), electricity demand arising from non-renewable sources such as coal is the main driver of the net carbon balance, accounting for 97%–99% of the carbon footprint of microalgae-based products. In this narrative, it is important to recognize that, although microalgae are often presented as a sustainable alternative, they cannot yet be considered entirely sustainable.

However, recent life cycle assessment (LCA) studies show that microalgae ingredients may have lower environmental impacts than conventional sources, especially proteins and carotenoids of animal or plant origin. Dutra et al. (2024) demonstrated that microalgae proteins have smaller environmental effects related to water footprint and land use change than conventional proteins, especially beef, which represents the worst-case scenario. Similarly, the production of β-carotene through conventional synthetic routes and microalgal processes did not show significant environmental risks; in contrast, palm oil showed higher impacts regarding climate change, ecotoxicity, and energy resources.

In another study, the production of *Spirulina* spp. showed a significantly lower environmental impact than beef production, presenting a decrease of up to 98% in greenhouse gas emissions (4.56 vs. 187.17 kg CO_2_-eq) and more than 99% in land occupation (0.25 vs. 116.95 m^2^ per equivalent of cultivation), regardless of the production system (Vannini and Achten, 2025). Ali et al. (2025) also observed that microalgae require significantly less land than traditional protein sources, using 95% less land than livestock and 60% less land than soy protein. Although the energy intensity of photobioreactors remains an obstacle, these results reinforce that microalgal products can outperform conventional ingredients in multiple environmental dimensions.

Furthermore, while natural products derived from microalgae offer proven benefits and promising applications, it is noteworthy that they are disruptive ingredients and are still in the intermediate stages of technological development (Bürck et al., 2024). This means that innovative foods, even when derived from natural or biological sources, are not exempt from environmental consequences and can have a greater environmental impact than conventional products, especially in terms of energy (Dutra et al., 2024). In this case, the challenge is to improve industrial processes and unit operations to build truly robust systems that make them economically competitive while reducing environmental impacts. In other words, production is not yet mature enough to achieve environmental and economic efficiency. Thus, the trajectory of microalgae lies between the potential for sustainability and the dystopian reality of technical, economic, environmental, commercial, and political limitations that need to be overcome.

However, adopting strategies such as integration into biorefineries, genetic optimization of strains, and using clean energy matrices can minimize ecological repercussions and reduce operating costs, thus increasing overall efficiency. In this context, integration into biorefineries has been identified as a potential alternative, as it allows the full use of biomass, converting it into multiple products such as proteins, lipids, and high-value bioactive compounds, within a circular bioeconomy model that maximizes resource efficiency and minimizes waste generation, improving environmental and economic viability (Barboza-Rodríguez et al., 2024). On the other hand, genetic optimization of strains and using emerging tools, such as biosensors coupled with artificial intelligence, results in increased productive performance and nutrient uptake efficiency (Hu et al., 2023; Ugya et al., 2025).

Likewise, the use of renewable energy is a promising alternative to face environmental and economic challenges, since, according to Dias et al. (2022), replacing coal-fired electricity with nuclear, hydroelectric, wind, biomass, or photovoltaic energy can reduce CO_2_ emissions by 94%–99%. Additionally, the carbon footprint associated with the dry biomass of *Chlorella vulgaris* and *Arthrospira platensis* can range from 16 to 29 kg CO_2_ e/year in countries that use clean sources to more than 2,000 kg CO_2_ e/year in coal-dependent countries (Dias et al., 2025). Therefore, using cleaner energy sources, in addition to minimizing the associated climate profile, also reduces dependence on fossil fuel-based electricity. In this sense, energy strategies bring microalgae-based products closer to the paradigm of true sustainability by linking climate action to cost reduction.

In particular, from an economic perspective, the energy required by conventional microalgae harvesting methods, such as centrifugation, flocculation, flotation, and filtration, as well as the initial investment in a bioreactor, is extremely costly. Consequently, the microalgae trade mainly focuses on high-value products, such as food supplements and nutraceuticals. The lack of more economical and efficient technologies prevents microalgae-based products from competing with conventional ingredients of plant and animal origin, limiting their insertion into other niches (Vázquez-Romero et al., 2022; Gao et al., 2024; Zhu et al., 2024). The truth is that the economic sustainability of microalgae in food design still faces long-standing barriers that require a systemic reconfiguration that transforms old obstacles into steps toward eco-efficiency.

An important pathway to reducing production costs in food applications is the integration of microalgae cultivation into biorefineries, since processing costs are distributed across several marketable compounds rather than concentrated in a single product. In a technical-economic analysis, Roles et al. (2021) demonstrated that the valorization of these compounds in the production chain can significantly reduce the minimum selling price of the product, making them more competitive in relation to conventional resources, especially when integrated with favorable geographic conditions and efficient cultivation systems.

Additionally, operational optimization tools that integrate weather forecasts into daily cultivation decisions have proven effective in reducing costs and increasing productivity. Gao et al. (2024) found that prediction-based dilution increased biomass productivity by 47% compared to batch cultivation and by 20% compared to fixed-rate dilution, thereby increasing resource utilization efficiency and reducing the energy intensity of harvesting operations. Furthermore, the selection of favorable geographic locations and climatic conditions can triple and quadruple the productivity of biomass and lipids in photobioreactors, respectively, while reducing the energy intensity of the process (Siqueira et al., 2020).

Other concrete strategies have been reported to reduce these costs, such as photoperiod modulation, since Maroneze et al. (2016) demonstrated energy savings of 33%–40.7% using short light/dark cycles of 0.50:0.50 s for biomass production and frequency regimes of 24–48 transitions per day for lipid production, respectively. In addition to reducing energy expenditure, specific photoperiod strategies also maximize the accumulation of target pigments, since according to do Nascimento et al. (2025), short photoperiods of 0.91:0.09 s increase the accumulation of pigments such as chlorophylls a and b, lutein, and β-carotene, while long photoperiods of 20:4 h promote the accumulation of compounds such as antheraxanthin. It is important to highlight that these studies were carried out on laboratory and pilot scales, requiring the development of technologies for the application of these methods on a commercial scale. These findings reinforce that economic feasibility is not solely dependent on technological advances, but also on strategic integration of geographic advantages and adaptive operational control.

Last but not least, in the social sphere, the application of microalgae in food design offers opportunities for innovation, such as in food fortification and the development of more nutritious and functional products. However, consumer acceptance of these products is a challenge, as psychological aspects and sensory properties are crucial factors in decision-making. In fact, studies show that the intense color (usually green) of microalgae, as well as their powdery texture and “marine” flavor and odor, make their application in food products difficult. In contrast, an alternative to neutralizing these sensory aspects is to add more processing, which would consequently increase costs and energy consumption. Furthermore, it is worth emphasizing that the lack of clear and standardized regulations for the use of microalgae in food formulations limits their consolidation in the market, as well as the lack of consumer knowledge about microalgae and their health benefits (Lafarga et al., 2021; Olsen et al., 2024; Zhu et al., 2024). These obstacles reinforce that environmental responsibility and the promise of contributing to global food security are not simple to achieve.

Furthermore, another challenge for the inclusion of microalgal biomass in food matrices is the consumer’s willingness to pay an additional price for ecological and innovation attributes. According to the meta-analysis by Li and Kallas (2021), consumers are willing to pay, on average, around 29.5% more for food products with sustainable characteristics. Cook et al. (2023) also observed that consumers are generally willing to pay moderately more for products with environmental and personal health-related credentials. Although this valuation varies greatly depending on the context and type of product. This demonstrates that there is recognition of the added value of environmental attributes, but it also highlights great heterogeneity between products, regions, and types of attributes, which implies that the appreciation for sustainability varies according to the context.

When comparing microalgae-derived alternatives with established products, such as fish oil supplements or conventional proteins, recent empirical studies in Europe point to positive effects of certain labels and credentials. Organic seals, Nutri-Scores, and vegan labels, for example, tend to increase purchase intentions and willingness to pay for products with microalgae. However, this impact varies significantly depending on the type of label, the reference product, and the consumer profile. Reviews and discrete choice studies also indicate that acceptance is still conditional, presenting marked differences between countries and market segments (Van der Stricht et al., 2023; Olsen et al., 2024; Hung et al., 2023).

However, beyond environmental appeal, many consumers still prioritize factors such as cost, taste, and familiarity, which limit the expansion of “premium” niches (Katare et al., 2023). In this sense, improving sensory properties is fundamental for the expansion of these products, as well as the development of technologies that contribute to the sustainability of the system. In short, there is a niche of consumers willing to pay for sustainable and innovative ingredients, including microalgae, but commercial consolidation requires that pricing, communication of nutritional and environmental benefits, and certifications be consistent with consumer expectations to compete with already established products.

Given this scenario, it is possible to affirm that the microalgae production in food design is a promising but still developing field, where enormous potential coexists with limitations that must be overcome. Full viability in all dimensions has not yet been achieved due to the various trade-offs. However, developing more economical closed or controlled systems for high-purity ingredients, combined with renewable energy and incentive policies, can lay the foundations on which the ideal of eco-efficiency can finally become reality. Microalgae can and should be seen as a strategic resource that can be consolidated through targeted investments and technological innovations, and cross the “valley of death” between scientific research and commercial success.

## 7 Conclusion

Microalgae are emerging as a crucial inflection point between global pressures for sustainability and opportunities for technological innovation. In recent decades, their nutritional and functional potential has been confirmed; however, translating this potential into tangible food inclusion still faces significant barriers. Analysis of the reviewed studies reveals that the barriers lie in the lack of systemic integration between scientific advances, the regulatory framework, and market strategies. The absence of standardized methodologies for biomass characterization, the high costs associated with subsequent processing, fragmented regulations, and consumer resistance—largely shaped by food neophobia—stand out as critical challenges.

In this context, the future agenda cannot be limited to incremental improvements but must prioritize integrated strategies that connect science, regulation, and social acceptance. Building consumer trust requires harmonized regulatory frameworks, transparent communication about safety, and accurate labeling. At the same time, technological efforts focused on sensory attributes must collaborate with gastronomy and marketing to transform perceptual barriers into opportunities for differentiation. Therefore, the effective incorporation of microalgae into global diets will depend on their ability to transcend the discourse of potential and materialize into sustainable, nutritious, economically accessible, and culturally acceptable products. Only through this convergence will microalgae consolidate themselves not as a distant promise, but as a structuring axis of the contemporary food bioeconomy.
